# High-Arsenic Acidic Wastewater Disposal in Non-Ferrous Smelting: A Critical Review of Fundamentals and Processes

**DOI:** 10.3390/ma19194200

**Published:** 2026-09-30

**Authors:** Xin Hong, Faxin Xiao, Chenyao Hao, Ganfeng Tu, Jipeng Liu, Xuwei Luo, Shuchen Sun

**Affiliations:** 1 School of Metallurgy, Northeastern University, Shenyang 110819, China; hongxin0622@163.com (X.H.); haocy1@mails.neu.edu.cn (C.H.); liujipeng@mails.neu.edu.cn (J.L.); 2410700@stu.neu.edu.cn (X.L.); sunsc@smm.neu.edu.cn (S.S.); 2 Key Laboratory for Ecological Metallurgy of Multimetallic Mineral (Ministry of Education), Northeastern University, Shenyang 110819, China; 3 Key Laboratory for Recycling of Nonferrous Metal Resources (Shenyang), Shenyang 110819, China

**Keywords:** high-arsenic acidic wastewater, sulfide precipitation, arsenic trisulfide colloid, competitive precipitation, nucleation and growth, non-ferrous smelting

## Abstract

**Highlights:**

- Sulfide precipitation is a major method for direct arsenic removal from strongly acidic HAAW.
- Four engineering bottlenecks constrain industrial sulfide precipitation.
- These bottlenecks are linked to three fundamental mechanistic knowledge gaps.
- Addressing thermodynamic, nucleation, and colloid gaps enables rational process design.
- Controlled-release sulfide sources and predictive models are key for next-generation treatment.

**Abstract:**

The co-smelting of complex gold concentrates with copper concentrates via the matte capturing process generates substantial high-arsenic acidic wastewater (HAAW) containing H_2_SO_4_ 50–60 g/L, As 15–30 g/L, and coexisting Cu, Pb, Zn, Cd, F^−^, and Cl^−^. This review critically evaluates treatment technologies, focusing on sulfide precipitation—a major method for direct arsenic removal under strong acidity. Lime neutralization, lime-ferric co-precipitation, scorodite, and sulfide precipitation are compared by principles and performance. Sulfide precipitation offers high efficiency, low sludge volume, and simultaneous heavy-metal removal; however, its application suffers from four bottlenecks: inaccurate dosing, low sulfur utilization, poor settleability due to colloidal As_2_S_3_, and insufficient residue stability. These are linked to three mechanistic knowledge gaps: thermodynamic equilibrium and competitive precipitation in multi-metal-sulfide-water systems, As_2_S_3_ nucleation and growth under supersaturation, and interfacial chemistry and coagulation of colloidal As_2_S_3_. These gaps span the entire reaction-nucleation-separation chain. This review bridges engineering and fundamental science, providing a theoretical basis for controllable sulfur release, selective metal recovery, and enhanced residue stability, guiding next-generation HAAW treatment.

## 1. Introduction

Arsenic is one of the most environmentally significant associated contaminants in non-ferrous metal smelting [1,2,3]. In copper, lead, zinc, and gold ores, arsenic commonly occurs as discrete associated minerals or through isomorphic substitution in mineral lattices and can be transferred into flue gas, solid residues, and liquid effluents during phase transformations in smelting processes [2]. Among these streams, high-arsenic acidic wastewater (HAAW) generated from flue-gas cleaning and sulfuric acid production is particularly difficult to treat because of its high acidity, high arsenic loading, high ionic strength, and the coexistence of multiple metals and halide ions [1,4,5]. Its treatment therefore involves complex acid-base equilibria, redox reactions, complexation, and precipitation processes [1,6,7].

The formation of HAAW essentially involves the transfer of arsenic from solid mineral phases to the gas phase, followed by its enrichment in the liquid phase [1,2]. During pyrometallurgical processing, arsenic-bearing sulfide minerals undergo thermal decomposition and oxidation, generating volatile or fine particulate arsenic-bearing species that enter the high-temperature flue gas [2]. After upstream dry dust collection, fine particles and gaseous arsenic species that are not completely captured subsequently enter the wet scrubbing system, where they are absorbed, dissolved, and progressively enriched in the circulating acidic liquor [1,2]. Continuous recirculation results in increasing concentrations of H_2_SO_4_, As, and other impurities, and a fraction of the circulating liquor must be discharged as HAAW once the impurity levels reach the process-control limits [1,4]. The composition of HAAW varies considerably among smelters and operating conditions because of differences in feed composition, smelting processes, flue-gas cleaning practices, and bleed strategies [1,2,4]. Published studies have reported H_2_SO_4_ and As concentrations of approximately 50–60 g/L and 15–30 g/L, respectively, in some representative HAAW streams; however, these ranges reflect specific operating conditions and should not be regarded as universal design parameters [1,4].

For such highly contaminated acidic wastewater, treatment performance cannot be evaluated solely by the removal of dissolved arsenic [1,8,9]. Engineering design must also consider residual acidity [1,4], H_2_S exposure risks [5,10], recovery of coexisting valuable metals [11,12], solid–liquid separation performance [13,14], residue generation, and the long-term environmental stability of arsenic-bearing solids [8,9]. HAAW treatment is therefore inherently a multi-objective process that integrates contaminant removal, resource recovery, and residue stabilization [1,12]. Within the current treatment framework, chemical precipitation remains the principal front-end process for removing high contaminant loads [1,4], whereas adsorption, membrane separation, ion exchange, and biological treatment are generally more suitable for advanced treatment or final polishing after substantial load reduction because of their limitations under high acidity, salinity, and contaminant concentrations [1,15].

Among the major chemical precipitation routes, sulfide precipitation has received sustained attention because it can directly convert and separate arsenic under strongly acidic conditions while reducing the need for pre-neutralization, limiting sludge generation [1,5,6], and facilitating the selective recovery of coexisting valuable metals [11,12]. With increasing mechanistic understanding, research has expanded from conventional optimization of the total sulfide dosage toward controlled sulfide release [16,17], enhanced gas–liquid mass transfer [10], selective sulfidation [11,12], micromixing regulation [13,14], particle nucleation and growth [11,13,14], and H_2_S emission control [10]. These developments indicate that the practical performance of sulfide precipitation depends not only on stoichiometry and thermodynamic driving forces [6,7] but also on arsenic valence state [7,11,18], sulfide-delivery mode [10,16,17], solution matrix [5,7], local mass-transfer and mixing conditions [10,13,14], particle evolution [11,13,14], and downstream residue-management strategies [8,9].

These coupled effects also account for several persistent challenges in the industrial application of sulfide precipitation: (1) variations in the As(III)/As(V) ratio, competitive reactions with coexisting metals, and nonproductive sulfur losses through volatilization and oxidation make the actual sulfide demand difficult to predict accurately [7,11,18]; (2) incomplete sulfide utilization and the formation and release of H_2_S under strongly acidic conditions increase safety-control requirements [5,10]; (3) high local supersaturation readily induces the formation of numerous fine As_2_S_3_-rich particles, which can form stable colloidal suspensions through interfacial charge interactions and thereby impair settling and filtration [13,14,18]; and (4) the resulting arsenic sulfide residues are often amorphous or poorly crystalline, leaving uncertainties regarding their long-term oxidation, dissolution, and phase-transformation behavior [8,9,18]. Previous reviews have systematically addressed dirty-acid treatment [1,5], metal sulfide precipitation, sulfide-source selection, particle aggregation [5,6], and Cu/As separation [12]. However, these issues have generally been discussed independently at different scales, and a systematic analysis integrating their interrelationships across the entire “reaction-particle formation-solid–liquid separation-residue stabilization” sequence remains limited [1,5,6,12].

### 1.1. Review Methodology and Scope

This review adopts a structured literature-search and evidence-evaluation approach tailored to a critical review rather than a formal systematic review or meta-analysis. Relevant studies were identified primarily through Web of Science Core Collection, Scopus, ScienceDirect, Google Scholar, and China National Knowledge Infrastructure (CNKI), covering the fields of environmental engineering, hydrometallurgy, extractive metallurgy, colloid science, and reaction engineering. The main literature search covered the period from 2000 to July 2026. Earlier representative and foundational studies were also retained where necessary to support discussions of thermodynamics, sulfide acid-base equilibria, classical nucleation theory, colloid interfacial chemistry, heterogeneous reaction engineering, and long-term solid-phase stability.

The search strategy combined subject-specific and process-related terms. The principal English keywords included “high-arsenic acidic wastewater”, “dirty acid”, “smelting waste acid”, “arsenic sulfide precipitation”, “metal sulfide precipitation”, “competitive sulfidation”, “H_2_S”, “NaHS”, “FeS”, “ZnS”, “scorodite”, “arsenic sulfide sludge”, “nucleation”, “particle growth”, “settling”, “zeta potential”, “XRD”, “leaching”, and “residue stabilization”. Boolean operators such as AND and OR were used to construct topic-specific search combinations. For Chinese-language literature, corresponding search terms including “high-arsenic acidic wastewater”, “smelting waste acid”, “arsenic sulfide precipitation”, “sulfide precipitation”, “competitive sulfidation”, “scorodite”, “arsenic sulfide colloid”, “nucleation and growth”, “flocculation and settling”, and “residue stabilization” were additionally used. Citation tracking of recent representative reviews and key primary studies was also performed to identify earlier publications of mechanistic or engineering relevance.

Literature selection focused on treatment performance, underlying mechanisms, engineering applicability, and the environmental stability of arsenic-bearing residues in HAAW systems. Priority was given to studies that: (i) investigated real HAAW or strongly acidic synthetic matrices representative of non-ferrous smelting waste acid in terms of acidity, arsenic concentration, and major coexisting components; (ii) clearly reported arsenic concentration and/or valence state, acidity or pH, sulfide reagent type, reagent ratio, and major reaction conditions; (iii) provided continuous, pilot-scale, or industrial operating data; and/or (iv) directly characterized precipitates or residues using methods such as XRD, XPS, SEM-EDS, particle-size analysis, zeta-potential measurements, or leaching tests. Studies focusing primarily on low-concentration arsenic removal from drinking water, showing limited relevance to acidic non-ferrous smelting wastewater, lacking key operating parameters, or providing insufficient evidence for the engineering or mechanistic issues discussed in this review were not used as the principal basis for comparison.

Because different types of studies address different scientific and engineering questions, this review does not apply a single hierarchy of evidence strength. Instead, the relative weight of evidence was considered according to the purpose of the analysis. Industrial, pilot-scale, and continuous studies were used primarily to assess engineering feasibility, operational stability, and scale-up applicability. Batch tests using real HAAW were used mainly to evaluate treatment performance under complex matrix conditions, whereas controlled experiments using synthetic solutions were used primarily to elucidate thermodynamic equilibria, reaction kinetics, nucleation and growth behavior, and particle interfacial mechanisms. Owing to the substantial heterogeneity among published studies in feed composition, As(III)/As(V) ratio, free acidity, sulfide source, S/As ratio, reaction time, solid–liquid separation method, and leaching assessment protocol, removal efficiencies and other performance metrics were not statistically pooled. Instead, the literature was synthesized through qualitative comparison and mechanism-oriented analysis.

### 1.2. Objective, Distinctive Contribution, and Conceptual Framework

The objective of this review is to explain why apparently favorable equilibrium chemistry does not automatically translate into controllable industrial HAAW treatment. The central conceptual proposition is that the four engineering bottlenecks are coupled consequences of incomplete understanding across a reaction-particle-separation-aging chain: feed speciation and sulfide delivery determine competitive reaction and local supersaturation [6,13]; local supersaturation and transport determine nucleation and growth [13,14]; particle structure and interfacial chemistry determine aggregation and separation [6,14]; and phase composition, crystallinity, and environmental exposure determine residue aging and arsenic leachability [8,9,19]. Thus, treatment performance cannot be predicted from removal efficiency or equilibrium constants alone.

The distinctive contribution is therefore not another catalog of treatment technologies. Instead, this review (i) maps engineering failures to three mechanistic knowledge gaps, (ii) distinguishes rate-controlling phenomena for soluble sulfide, gaseous H_2_S, and slow-release solid sulfides, (iii) links particle formation and XRD/solid-state characterization to residue stability, and (iv) separates evidence that is well established from mechanisms that remain conditional in high-ionic-strength, multicomponent HAAW. Table 1 compares this scope with representative recent reviews.

## 2. Arsenic in Non-Ferrous Smelting: Occurrence, Migration and Hazards

### 2.1. Process-Relevant Properties and Speciation of Arsenic

For HAAW treatment, the arsenic properties most relevant to process performance include oxidation state, aqueous speciation [7,18,21], volatilization and transport behavior during pyrometallurgical processing [2,22], and sulfidation reactivity [7,11,18]. In aqueous systems, arsenic occurs predominantly in the As(III) and As(V) oxidation states [18,21]. Under strongly acidic conditions, As(III) exists mainly as undissociated H_3_AsO_3_, whereas As(V) occurs predominantly as highly protonated species such as H_3_AsO_4_ and H_2_AsO_4_^−^ [18,21]. Their exact distributions are further influenced by pH, redox potential, ionic strength, and complexation [7,21]. These speciation differences directly affect subsequent sulfide precipitation because As(III) and As(V) differ markedly in sulfide stoichiometric demand, reaction pathways, and reduction kinetics [7,11,18].

During pyrometallurgical processing, thermal decomposition and oxidation of arsenic-bearing sulfides can generate volatile arsenic species as well as As_2_O_3_/As_4_O_6_^−^ containing vapors or fine particulates, facilitating the transfer of arsenic from the furnace feed to the flue gas and subsequently to the wet gas-cleaning liquor [2,22]. After entering the aqueous phase, As(III) can react with sulfide species such as H_2_S and HS^−^ in acidic sulfide-bearing systems to form sparingly soluble As_2_S_3_ precipitates, whereas the sulfidation of As(V) generally also involves a reduction step [7,11,18]. These speciation and transformation characteristics directly govern arsenic migration [2,22], sulfide demand, and precipitation behavior in HAAW [7,11,18]. Accordingly, this review focuses on arsenic speciation and transformations directly relevant to treatment processes, rather than on general physicochemical properties such as allotropy, density, and melting point, which are of limited relevance to HAAW treatment.

### 2.2. Mineral Occurrence and Sources of Arsenic

Arsenic is a ubiquitous element in the Earth’s crust, with an average abundance estimated at approximately 1.7–2.0 mg/kg [23,24]. This background concentration varies considerably depending on rock type and geological setting, with some arsenic-rich formations containing substantially higher levels [24]. The mineralogy of arsenic is exceptionally diverse. To date, more than 760 distinct arsenic-bearing mineral species have been identified [24,25]. From a compositional perspective, arsenate minerals constitute approximately 60% of known arsenic minerals, sulfides and sulfosalts account for about 20%, and the remaining 20% comprise arsenides, arsenites, oxides, silicates, and elemental arsenic [23,24]. Among the most industrially significant arsenic minerals are arsenopyrite (FeAsS), which is the most common arsenic-bearing mineral and an important source of arsenic in many ore deposits [24,26]. Realgar (As_4_S_4_) and orpiment (As_2_S_3_) are arsenic sulfides commonly associated with hydrothermal mineralization [24]. Enargite (Cu_3_AsS_4_) and tennantite (Cu_12_As_4_S_13_) are important Cu–As-bearing minerals frequently encountered in copper ores [24,27]. Scorodite (FeAsO_4_·2H_2_O) is a ferric arsenate that commonly occurs as a secondary oxidation product of arsenic-bearing sulfide minerals [24].

As a chalcophile element, arsenic exhibits a strong affinity for sulfur [24]. Beyond forming its own discrete mineral species, arsenic commonly occurs in pyrite and other sulfide minerals through lattice substitution or incorporation into the sulfide structure [27,28]. This substitution behavior contributes to the widespread association of arsenic with copper-, lead-, zinc-, and gold-bearing mineral systems [27,29]. The magnitude of this association is substantial: approximately 15% of global copper resources have been reported to exhibit As:Cu mass ratios as high as 1:5 [30]. For gold resources, it has also been reported that approximately 5% may contain As:Au ratios reaching 2000:1 [31]. Consequently, arsenic is inevitably introduced into metallurgical processing streams as an associated impurity whenever these ores are treated [27,29,32].

The global distribution and sources of arsenic are uneven and closely related to the occurrence and processing of arsenic-bearing mineral deposits [23,26,33]. In China, arsenic resources are concentrated primarily in the central-southern and western regions, and the Shimen realgar deposit in Hunan Province represents an important arsenic-bearing mineral deposit in China [23,33]. Commercial arsenic can be recovered from copper, gold, and lead smelter flue dust, from the roasting of arsenopyrite, and from the processing of arsenic-bearing minerals such as realgar, orpiment, and enargite [26,27]. The specific recovery route varies with country, ore type, and metallurgical process [23,26]. Arsenic has been recovered from realgar and orpiment in China, Peru, and the Philippines and from copper–gold ores in Chile, whereas arsenic is commonly associated with gold occurrences in Canada [23,26].

### 2.3. Formation Mechanism of HAAW

The formation of HAAW in non-ferrous metal smelting can be described as a continuous migration process of arsenic from the solid phase (ore minerals) → gas phase (volatile arsenic species) → liquid phase (acidic scrubbing liquor) [1,2]. This process determines the speciation and loading of arsenic entering the flue-gas cleaning system and provides an important basis for understanding the characteristics of HAAW and its subsequent treatment requirements [1,2].

During pyrometallurgical processing, arsenic-bearing sulfide minerals undergo thermal decomposition and oxidation at high temperatures (typically 1000–1300 °C) [2]. Their reaction pathways and volatile arsenic species are jointly influenced by temperature, oxygen potential, furnace type, and feed composition [2,34]. Taking arsenopyrite as an example, its decomposition reactions can be represented as follows [2,35]:

4FeAsS (s) → 4FeS (s) + As_4_ (g) (under reducing conditions) (1)

4FeAsS (s) + 10O_2_ (g) → 2Fe_2_O_3_ (s) + 4SO_2_ (g) + As_4_O_6_ (g) (under oxidizing conditions) (2)

These reactions are intended to illustrate possible transformation pathways of arsenopyrite under different oxidation conditions and do not fully represent the complex multiphase reaction system in actual smelting furnaces [2,35]. At high temperatures, arsenic may occur in the gas phase as several volatile species, including As_2_(g), AsS(g), AsO(g), and gaseous arsenic oxides such as As_4_O_6_, depending on temperature, oxygen potential, sulfur potential, and operating conditions [22,34,35]. Under relatively low-oxygen-potential conditions, as reported for the gas phase of the SKS oxygen bottom-blown copper smelting process, As_2_(g), AsS(g), and AsO(g) can be the predominant gaseous arsenic species, whereas more strongly oxidizing conditions favor the formation of arsenic oxides [34,35]. Therefore, arsenic transfer to the flue-gas phase should not be attributed solely to the volatilization of arsenic trioxide, because arsenic can enter the gas phase through multiple volatilization and gas-phase transformation pathways [22,34].

The partitioning of arsenic among flue gas, matte, and slag is also strongly process-dependent [2,34]. A study conducted under stable operating conditions of SKS bottom-blowing copper smelting reported that approximately 6%, 12%, and 82% of arsenic were distributed to the matte, slag, and gas phase, respectively [34]. Compared with flash smelting, bottom-blowing smelting can, under specific conditions, transfer a higher proportion of arsenic to the gas phase, whereas flash smelting may retain more arsenic in the matte and slag [34]. These differences are mainly related to oxygen potential, temperature, reaction intensity, mass-transfer conditions, and matte grade [34]. Therefore, the reported partitioning ratios should be regarded as representative results under specific operating conditions rather than universal constants [34].

After entering the flue gas, arsenic-bearing components pass sequentially through waste-heat recovery and dry dust-removal units [1,22]. Larger arsenic-bearing particles can be captured in waste-heat boilers, electrostatic precipitators, or bag filters, whereas some fine particles and volatile arsenic-bearing species may continue into the wet gas-cleaning section of the sulfuric acid system [1,22]. As the flue-gas temperature decreases, some volatile arsenic species may undergo oxidation, condensation, or particle formation and ultimately enter the scrubbing system as fine particulates or gaseous components [22].

During wet scrubbing, the flue gas is typically contacted with dilute sulfuric acid or water to reduce its temperature, remove residual particulates, and eliminate SO_3_, HCl, HF, and other impurities [1,4]. After entering the scrubbing liquor, arsenic oxides can form aqueous arsenic species dominated by As(III), while a fraction may be further oxidized to As(V) under oxidizing conditions [1,21]. Meanwhile, metals, chlorides, fluorides, and other impurities in the flue gas are also transferred to the circulating liquor [1,4]. With continuous recirculation, H_2_SO_4_, As, and other impurities progressively accumulate [1,4]. When their concentrations reach the process-control limits, a portion of the circulating liquor must be discharged as HAAW [1,4]. Figure 1 illustrates the formation of HAAW in the flue-gas cleaning section of a sulfuric acid plant.

The composition of HAAW varies considerably among smelters and operating conditions because of differences in feed composition, smelting processes, flue-gas cleaning practices, and bleed strategies [1,4]. Some representative HAAW streams have been reported to contain approximately 50–60 g/L H_2_SO_4_ and 15–30 g/L As, together with coexisting species such as Cu, Pb, Zn, Cd, F^−^, and Cl^−^ [1,4]. These values represent typical wastewater characteristics under specific operating conditions and should not be regarded as fixed compositions or universal design parameters applicable to all HAAW systems [1,4]. The high acidity, high ionic strength, and coexistence of multiple metals and anions make the chemical behavior and treatment of HAAW substantially more complex than those of conventional arsenic-containing wastewater [1,7].

### 2.4. Toxicity and Environmental Hazards of Arsenic

#### 2.4.1. Human Health Effects

Arsenic is classified as a Group 1 carcinogen by the International Agency for Research on Cancer (IARC), with established links to skin, lung, and bladder cancers [36]. The primary routes of human exposure are through contaminated drinking water, food chain accumulation, inhalation of arsenic-bearing dusts, and dermal contact [37,38,39]. For the general population, food and drinking water constitute the dominant exposure pathways [38,39,40].

The toxicity of arsenic is strongly dependent on its chemical form [41,42,43]. In general, inorganic arsenic species are substantially more toxic than organic arsenic compounds, and trivalent arsenic (As(III)) is generally more toxic than pentavalent arsenic (As(V)), with approximately 60-fold greater potency reported in some biological assays [41,42,43,44]. The mechanistic basis for this differential toxicity lies in the strong affinity of As(III) for sulfhydryl (thiol, -SH) groups present in proteins and enzymes [41,42,43]. By binding to these critical sulfhydryl groups, As(III) inhibits the activity of numerous thiol-dependent enzymes, thereby disrupting cellular metabolism and energy production [41,42,43]. As(V), in contrast, competes with phosphate in biochemical reactions, interfering with ATP synthesis and other phosphorylation processes [41,42,43].

Acute high-dose arsenic exposure manifests as gastrointestinal and circulatory system damage: increased vascular permeability, fluid loss, hypotension, and in severe cases, hemorrhagic gastroenteritis and multi-organ failure [41]. Chronic low-dose exposure, however, presents a more insidious hazard [41]. Long-term consumption of arsenic-contaminated water or food can lead to cumulative arsenic body burden and chronic arsenic poisoning, characterized by skin pigmentation changes, hyperkeratosis (particularly on palms and soles), peripheral neuropathy, liver and kidney damage, non-cirrhotic portal hypertension, and in extreme cases, peripheral gangrene—the condition historically known as “blackfoot disease” [39,41,42]. Urinary arsenic is widely used as a biomarker of recent arsenic exposure, but its interpretation should consider arsenic speciation and dietary sources that may influence total urinary arsenic concentrations [39]. Hair arsenic can provide complementary information on longer-term exposure; however, external contamination and methodological variability limit its use as a stand-alone diagnostic indicator [45].

#### 2.4.2. Environmental Effects

Arsenic contamination in the environment arises from both natural and anthropogenic sources [40,46,47]. Natural sources include mineral weathering, volcanic activity, and geothermal processes; anthropogenic sources encompass non-ferrous metal mining and smelting, coal combustion, chemical manufacturing, pesticide production and application, and various industrial activities including ceramics, glass production, wood preservation, textiles, tanning, pharmaceuticals, paints, paper manufacturing, and fertilizer production [40,46,47]. Water contamination from arsenic is primarily associated with mining, smelting, and chemical industry effluents, as well as leachate from waste slag deposits [40,46,47]. In natural waters, arsenic typically exists as inorganic arsenite and arsenate species [40,46]. Arsenic contamination reduces drinking water safety, harms aquatic organisms, and can enter the soil-crop system through irrigation with contaminated water [40,46,48,49].

Soil contamination arises from arsenic-containing pesticide application, mine drainage, industrial waste slag, smelting dust deposition, and coal combustion fly ash [40,46,47]. Background soil arsenic concentrations are typically on the order of approximately 5–10 mg/kg, but anthropogenic activities can elevate this substantially [46,50]. According to China’s 2014 National Soil Pollution Survey Bulletin, the exceedance rate for arsenic among inorganic pollutants was 2.7% [51]. Mining and industrial areas represent important zones of concern for arsenic and other potentially toxic elements [50,51]. Arsenic entering soil may be adsorbed by minerals, organic matter, and iron/aluminum oxides [46,52]; however, changes in pH, redox conditions, or microbial activity can trigger its re-release, inhibiting microbial activity, reducing soil fertility, and entering the food chain through crop uptake [52,53].

Atmospheric contamination from non-ferrous smelting and coal combustion releases arsenic-bearing particulates or gaseous arsenic compounds [40,46,47]. These materials deposit onto soil and water bodies through dry and wet deposition [46,47,54], reducing air quality and posing respiratory and cardiovascular health risks [55,56]. Atmospheric deposition can also increase arsenic loading to terrestrial ecosystems [54], where arsenic exposure can adversely affect crop and vegetation growth and plant physiological functions [53,57].

Overall, arsenic pollution exhibits cross-media migration and coupled transformation characteristics, propagating through water, soil, atmosphere, and the food chain to create long-term cumulative ecological and health risks [40,47,49,53,54,56]. Figure 2 schematically illustrates the migration pathways of arsenic from various sources through environmental media to human exposure.

This cross-media migration of arsenic underscores the importance of integrated management strategies focused on source control at smelting operations, which can serve as important anthropogenic source points of arsenic contamination [40,47,54].

## 3. Overview of Treatment and Stabilization Technologies for HAAW

The treatment of HAAW generally follows a two-stage philosophy: primary removal at the front end, where the bulk of arsenic and heavy metals are precipitated from the highly acidic liquor, followed by polishing treatment at the back end to reduce residual contaminants to acceptable levels. Primary removal is predominantly accomplished by chemical precipitation methods, which exploit the formation of sparingly soluble arsenic compounds to effect solid–liquid separation [1,4,20]. Physical methods—including adsorption, membrane separation, and ion exchange—and biological methods are also established approaches for arsenic or heavy-metal removal from water and wastewater [44,58]. However, because raw HAAW is characterized by extreme acidity and high contaminant loads, these methods are generally more suitable as supplementary or polishing treatments for pretreated or lower-strength streams than for direct treatment of raw HAAW [1].

This section provides a critical overview of the principal treatment technologies, with emphasis on their fundamental chemistry, operational characteristics, engineering applicability, and inherent limitations. Detailed treatment of sulfide precipitation—a particularly important technology for direct arsenic removal under strongly acidic conditions—is deferred to Section 4.

### 3.1. Lime Neutralization

Lime neutralization is one of the oldest and most widely applied methods for treating acidic metallurgical wastewaters [1,4,59,60]. The fundamental principle is the addition of lime (CaO) or hydrated lime (Ca(OH)_2_) to neutralize acidity and precipitate arsenic, fluoride, and heavy metals as calcium salts, hydroxides, or co-precipitates [1,4,59,60].

#### 3.1.1. Reaction Chemistry

The neutralization process proceeds through several reactions. Sulfuric acid in the wastewater is first neutralized [1,4,59,60,61]:

Ca(OH)_2_ (s) + H_2_SO_4_ (aq) → CaSO_4_·2H_2_O (s) (3)

This neutralization reaction commonly results in gypsum (CaSO_4_·2H_2_O) precipitation [1,4,59,60]. As the pH rises, arsenite and arsenate can be removed through the formation of calcium arsenite, calcium arsenate, and associated co-precipitates [1,60,62,63,64]. The following equations are simplified stoichiometric representations of these precipitation reactions:

3Ca(OH)_2_ (s) + 2H_3_AsO_3_ (aq) → Ca_3_(AsO_3_)_2_ (s) + 6H_2_O (l) (4)

3Ca(OH)_2_ (s) + 2H_3_AsO_4_ (aq) → Ca_3_(AsO_4_)_2_ (s) + 6H_2_O (l) (5)

For As(III)-bearing Ca–As sludge, mineralogical and thermodynamic studies have shown that newly formed amorphous calcium arsenite phases, such as CaHAs(III)O_3_·xH_2_O, can form during lime stabilization, and their formation and transformation significantly influence the leaching behavior of As(III) [62]. In addition to As(III), the phase composition and stability of Ca–As precipitates in As(V) systems are also strongly affected by the Ca/As molar ratio and solution pH [63,64,65].

At 25 °C in the Ca^2+^—AsO_4_^3−^—H_2_O system, several calcium arsenate phases may form depending on the solution chemistry, including CaHAsO_4_·H_2_O(s), hydrated Ca_3_(AsO_4_)_2_·4H_2_O(s) phases, Ca_5_(AsO_4_)_3_OH(s), and Ca_4_(OH)_2_(AsO_4_)_2_·4H_2_O(s) [63,64,65]. The thermodynamic model developed by Zhang et al. further showed that the Ca/As molar ratio and pH jointly determine the stability fields of different calcium arsenate phases. When Ca/As < 2, the stability fields of CaHAsO_4_·H_2_O and Ca_3_(AsO_4_)_2_·4H_2_O decrease with increasing pH, whereas Ca_4_(OH)_2_(AsO_4_)_2_·4H_2_O can form when Ca/As > 2 [65]. Thus, the Ca/As molar ratio and pH not only govern the types of Ca–As solids formed but also influence the dissolution and stability of the resulting precipitates.

The solubilities of different calcium arsenate phases vary markedly. Among them, Ca_5_(AsO_4_)_3_OH has a relatively low reported solubility product, K_sp_ ≈ 10^−40.12^ [64]. It should be noted that the thermodynamic stability of different calcium arsenate phases cannot be directly compared solely on the basis of their K_sp_ values because their dissolution reactions and stoichiometries differ. Their stability fields are also jointly influenced by pH, the Ca/As molar ratio, and component activities [63,64].

Based on these phase-formation and dissolution characteristics, Table 2 summarizes the reported arsenic concentrations in leachates and solubility-product data for representative calcium arsenate phases [60,64], enabling further comparison of the leaching behavior and stability of different Ca–As solids.

As shown in Table 2, arsenic leachability varies markedly among different calcium arsenate phases. Reported arsenic concentrations in the leachates range from 0.44 mg/L for Ca_4_(OH)_2_(AsO_4_)_2_·4H_2_O to 3464 mg/L for Ca_3_(AsO_4_)_2_·3H_2_O [60,64]. These results indicate that the leaching behavior of arsenic from different Ca–As phases is closely related to phase identity, hydration state, and structural characteristics, and cannot be assessed solely on the basis of the bulk Ca/As molar ratio. Therefore, the phase composition, solution conditions, and relevant process parameters should be considered collectively when evaluating the stability and potential environmental risks of Ca–As residues [60,64]. These differences further highlight the importance of controlling precipitation conditions and operating parameters during lime neutralization. Figure 3 illustrates the typical process flow of lime neutralization for HAAW treatment.

#### 3.1.2. Limitations and Environmental Concerns

Despite its low cost, operational simplicity, and widespread industrial use, lime neutralization suffers from several critical limitations when applied to HAAW. First, the process generates large volumes of gypsum-bearing sludge, which typically contains arsenic distributed heterogeneously throughout the mass [1,4,59,60]. Second, the Ca–As precipitates formed may exhibit limited long-term stability under changing storage and environmental conditions. Studies have shown that atmospheric CO_2_ can induce transformation of calcium arsenates to calcium carbonate, releasing arsenic in the process [67]. Dry-wet cycling, freeze–thaw cycling, and pH variations during storage can further promote phase transformation and arsenic remobilization from Ca–As residues [67,68,69]. Third, when applied alone, lime neutralization may not reliably reduce arsenic concentrations to stringent discharge levels, particularly for high-arsenic streams [1,59,60]. Fourth, the process can require substantial lime addition, increasing reagent consumption and sludge generation [1,59,60].

For these reasons, lime neutralization is now more appropriately viewed as a pre-neutralization or preliminary arsenic removal step, rather than a standalone treatment. In practice, it can be combined with sulfide precipitation (for selective arsenic removal) or ferric co-precipitation (for enhanced arsenic stabilization) to achieve both high removal efficiency and long-term residue stability [1,4,6,20,70].

#### 3.1.3. High-Density Sludge Process

The high-density sludge (HDS) process is an improved form of conventional lime neutralization. Its core principle is to recycle a portion of the neutralized sludge to the reaction system as seed sludge and a substrate for precipitate growth, followed by thorough mixing with lime [71,72]. This promotes the deposition and growth of newly formed precipitates on existing solid surfaces, while facilitating particle aggregation and densification, thereby increasing sludge solids content and improving settling performance [71,72].

For acid mine drainage treatment, the HDS process has been reported to produce sludge containing approximately 25–30% solids [71,72]. In addition, compared with conventional lime neutralization, HDS can improve lime utilization efficiency and reduce the volume of wet sludge requiring handling and disposal [71,72]. Therefore, this process is particularly suitable for large-scale acidic wastewater treatment where sludge handling and disposal represent a substantial operational burden.

However, the actual performance enhancement achieved by the HDS process depends on feed composition, sludge recycle ratio, lime addition strategy, and overall process configuration [71,72]. For HAAW with complex compositions and high acidity and arsenic concentrations, sludge densification, lime consumption, and solid–liquid separation performance should therefore be evaluated according to the specific wastewater characteristics and operating conditions. Accordingly, HDS should primarily be regarded as an engineering enhancement of conventional lime neutralization, and its performance benefits reported mainly for acid mine drainage systems should not be directly generalized to HAAW without system-specific validation [71,72].

### 3.2. Lime-Ferric Co-Precipitation

Lime-ferric co-precipitation builds upon lime neutralization by introducing iron salts (typically ferric sulfate or ferric chloride) to enhance arsenic removal through adsorption, surface complexation, and co-precipitation with iron (oxyhydr)oxides [1,70,73,74,75]. In some staged treatment processes, ferrous sulfate is used instead of ferric salts and is subsequently oxidized by aeration to generate Fe(III)-bearing precipitates [76,77]. 

#### 3.2.1. Reaction Mechanism

In the first stage, lime is added for neutralization and the initial formation of calcium-containing arsenic precipitates, following a reaction pathway generally consistent with that described in Section 3.1. In the second stage, ferric salts are added while the pH is further adjusted. As the pH increases, Fe(III) undergoes hydrolysis to form amorphous or poorly crystalline iron (oxyhydr)oxides, commonly represented as FeOOH or Fe(OH)_3_. These solids possess high specific surface areas and abundant surface hydroxyl groups, which can adsorb As(V) and As(III) species through ligand-exchange reactions, forming inner-sphere surface complexes [73,74,75]. The surface complexation reactions can be simplified as follows:

≡ FeOH + H_2_AsO_4_^−^ (aq) → ≡ FeHAsO_4_^−^ + H_2_O (l) (6)

≡ FeOH + H_3_AsO_3_ (aq) → ≡ FeH_2_AsO_3_ + H_2_O (l) (7)

Here, the symbol ≡ denotes a surface hydroxyl site on iron (oxyhydr)oxides.

In addition to surface adsorption, arsenic can also be incorporated into newly formed iron (oxyhydr)oxide solids through co-precipitation, occlusion, or structural incorporation during Fe(III) hydrolysis and precipitation [75,78]. For As(V), ferric arsenate-type solids may also form under suitable conditions, which can be represented by the following simplified reaction [73,78]:

H_3_AsO_4_ (aq) + Fe(OH)_3_ (s) → FeAsO_4_ (s) + 3H_2_O (l) (8)

#### 3.2.2. Three-Stage Lime-Ferric Process

A refined three-stage process has been developed for treating high-arsenic, high-cadmium wastewaters [1,76,77]. In the first stage, lime is added to pH 2 to precipitate relatively pure gypsum, which can potentially be utilized as a byproduct [76,77]. In the second stage, lime addition to pH 5–6 with ferrous sulfate addition (Fe/As molar ratio ≈ 4:1) and aeration removes the bulk of arsenic [76,77]. In the third stage, further lime addition to pH 9 with additional ferrous sulfate and aeration removes residual arsenic and other heavy metals [76,77]. Using this approach, arsenic concentrations as high as 14,740 mg/L have been reduced to 0.25 mg/L, with cadmium below detection limits [76,77]. The process flow of this representative three-stage configuration is illustrated in Figure 4.

A more recent optimized lime–iron salt process used gypsum seeding at pH 1.2, followed by H_2_O_2_ oxidation and arsenic removal at pH 8 with an Fe/As molar ratio of 4.5, reducing the residual As concentration to 0.12 mg/L [79]. In addition to three-stage configurations, a two-stage continuous lime–Fe(III) co-precipitation process has also been investigated. At an Fe/As molar ratio of 4, sequential operation at pH 4 and pH 8 reduced the As(V) concentration from 1418 mg/L to 0.008 mg/L, corresponding to a removal efficiency of >99.99% [80]. The representative operating conditions and treatment performances of the two-stage, three-stage, and optimized lime–iron co-precipitation processes are compared in Table 3.

#### 3.2.3. Limitations

Although lime-ferric co-precipitation generally achieves higher arsenic removal efficiency than lime treatment alone, its engineering application is still constrained by reagent consumption, sludge generation, and residue stability. The process requires relatively high dosages of lime and iron salts, resulting in elevated effluent pH that may require readjustment before discharge or subsequent treatment, while also generating large quantities of arsenic-bearing sludge containing gypsum and iron phases [1,59,70,76,79].

In addition, ferric arsenate-type precipitates formed under non-hydrothermal conditions are often amorphous or poorly crystalline and may exhibit lower stability than well-crystallized scorodite (FeAsO_4_·2H_2_O), with potential arsenic release under changing environmental conditions [78,81,82]. Therefore, in addition to aqueous-phase arsenic removal efficiency, the long-term stability of the residues should be evaluated based on solid-phase composition, crystallinity, and leaching behavior.

### 3.3. Scorodite Precipitation

Scorodite (FeAsO_4_·2H_2_O) is a crystalline ferric arsenate mineral with low solubility and a relatively high arsenic content (approximately 32 wt% As) [78,83,84]. Owing to its low arsenic leachability and favorable environmental stability, scorodite is considered one of the important solid phases for the stabilization and disposal of arsenic [4,78,83,84,85]. Previous studies have shown that well-crystallized scorodite can exhibit As leaching concentrations below 5 mg/L [84,86].

#### 3.3.1. Synthesis Conditions

The formation of scorodite is sensitive to pH, temperature, Fe/As ratio, and iron source, which distinguishes it from the amorphous ferric arsenate formed during conventional lime-ferric treatment [78,83,85,86,87]. In general, acidic conditions favor scorodite formation and stability, although the specific stability range and optimum synthesis conditions depend on solution composition and synthesis route [78,83,85,86,87,88,89]. In one hydrothermal synthesis study, when the initial pH was 1.0–2.0, the Fe/As molar ratio was 0.5–3.0, and the reaction temperature was 105–175 °C, the arsenic precipitation efficiency exceeded 80%, and the As leaching concentration of the resulting scorodite was below 5 mg/L [86]. Under optimized conditions (pH 1.5, Fe/As = 1.0, 150 °C), octahedral scorodite particles with sizes of approximately 20 μm were obtained, with an As leaching concentration as low as 0.08 mg/L [86]. In addition, crystalline scorodite can be produced by atmospheric oxidative precipitation at approximately 90–95 °C [88,89]. Table 4 summarizes typical operating conditions and performance of scorodite precipitation processes.

#### 3.3.2. Multi-Stage Scorodite Process

To shorten the relatively long reaction time required for conventional scorodite precipitation, multistage precipitation processes have been developed. In one study on smelting waste acid, the pH was gradually increased from 1.1 to 3.8 over five stages, combined with air sparging and scorodite seeding. An arsenic precipitation efficiency of 97.1% was achieved, while copper loss was only 9.1%, indicating that the process can provide effective Cu-As separation under specific conditions [90].

#### 3.3.3. Challenges for Industrial Application

Although scorodite exhibits favorable arsenic immobilization performance, its industrial application remains constrained by reaction-condition control, equipment requirements, and energy consumption [83,86,87,88,89]. Hydrothermal synthesis requires relatively high temperatures and pressure- and corrosion-resistant equipment, whereas atmospheric-pressure processes generally require precise control of pH, oxidation rate, and seeding conditions [83,86,87,88,89]. Under specific conditions, coexisting Na^+^ and K^+^ may also promote the formation of jarosite-type phases, such as natrojarosite and jarosite, which compete with scorodite crystallization and thereby affect product composition and arsenic immobilization performance [83,91]. In addition, strongly acidic operating conditions impose stringent requirements on equipment materials and corrosion control. Therefore, reducing the reaction temperature and reaction time while enhancing selective crystallization in complex HAAW systems remains an important direction for the engineering development of scorodite processes [83].

### 3.4. Physical Methods

#### 3.4.1. Adsorption

Adsorption exploits the high surface area and surface functional groups of adsorbent materials to capture and immobilize arsenic species from aqueous solution [15,92]. The process involves physical adsorption (van der Waals forces), chemical adsorption (surface complexation), or ion exchange at the adsorbent surface [15,92]. Arsenic removal by adsorption is strongly influenced by arsenic speciation: As(V) is generally more readily adsorbed than As(III) because As(V) exists as anionic species (H_2_AsO_4_^−^, HAsO_4_^2−^) across a wide pH range, while As(III) is predominantly uncharged (H_3_AsO_3_) under neutral to weakly acidic conditions and therefore less amenable to electrostatic attraction [15,92].

Recent research has focused on advanced adsorbent materials including iron-modified biochars, nanoscale iron oxides, metal–organic frameworks (MOFs), and functionalized porous materials [92,93]. However, for HAAW applications, adsorption faces fundamental limitations: the extreme acidity and high ionic strength of the wastewater may adversely affect the stability and adsorption performance of some adsorbent materials; the high arsenic concentration can rapidly consume available adsorption capacity; and competing ions (sulfate, chloride, heavy metals) reduce selectivity and capacity [1,15,92]. Adsorption is therefore more suitable for polishing pretreated or relatively low-concentration arsenic-bearing streams than for primary treatment of raw HAAW [1,15,92].

#### 3.4.2. Membrane Separation

Membrane processes—including microfiltration (MF), ultrafiltration (UF), nanofiltration (NF), and reverse osmosis (RO)—separate contaminants based on size exclusion and charge interactions [15,94]. NF and RO are most relevant for arsenic removal, as they can effectively reject dissolved arsenic species [15,93,94]. However, membrane processes face severe challenges when applied to HAAW: the extreme acidity may constrain the chemical stability and compatibility of membrane materials; high suspended solids and salt concentrations can promote membrane fouling; high osmotic pressures require substantial energy input; and the disposal of concentrated brine streams presents additional environmental burdens [1,15,93,94]. Membrane processes are primarily applicable to pretreated or diluted streams rather than direct treatment of raw HAAW [1,15,94].

#### 3.4.3. Ion Exchange

Ion exchange utilizes solid resins bearing functional groups that exchange ions with the solution phase [15,94]. Strong-base anion exchange resins are most effective for As(V) removal, as As(V) exists as anionic species amenable to exchange [15,94]. However, As(III) removal is poor because uncharged H_3_AsO_3_ predominates under neutral to weakly acidic conditions [15,94]. In HAAW applications, the high concentrations of competing anions (sulfate, chloride, fluoride) can substantially reduce the effective exchange capacity and increase regeneration requirements [1,94]. The extreme acidity may also constrain resin stability and regeneration performance [1,94]. Like adsorption and membrane processes, ion exchange is best suited for polishing applications rather than primary HAAW treatment.

### 3.5. Biochemical Methods

Biochemical methods leverage microorganisms, algae, or plants to adsorb, accumulate, or transform arsenic and heavy metals [95,96]. The mechanisms include non-metabolic biosorption (involving cell wall functional groups such as carboxyl, hydroxyl, and amino groups) and metabolically driven biotransformation (including As(III) oxidation, As(V) reduction, and arsenic methylation) [95,96].

While biochemical methods offer potential advantages, including relatively low cost and environmental compatibility [95,96], they are fundamentally limited in HAAW applications. The extreme acidity and high arsenic concentrations of raw HAAW create conditions that are generally unfavorable for many conventional biological treatment systems [1,96]; the long growth cycles and sensitivity to environmental conditions make process control difficult [95,96]; and the relatively slow kinetics may limit their applicability to the high-throughput requirements of industrial smelting wastewater treatment [1,96]. Biochemical methods are therefore more suitable for lower-strength and less acidic arsenic-bearing waters than for raw high-acid, high-arsenic HAAW [1,95,96].

### 3.6. Technology Comparison and Process Selection

HAAW treatment technologies differ markedly in arsenic removal efficiency, acidity preservation, sludge generation, residue stability, resource recovery potential, and engineering maturity. Table 5 provides a comprehensive comparison of the major treatment and stabilization technologies. It should be noted that substantial differences exist among studies in initial As concentration, acidity, arsenic speciation, coexisting components, operating conditions, solid–liquid separation methods, and analytical procedures. Therefore, the reported removal efficiencies and residue performance should not be directly ranked without considering these differences.

No single treatment technology can simultaneously optimize all performance criteria. For high-concentration, strongly acidic HAAW, sulfide precipitation is generally advantageous when acidity preservation, relatively low sludge generation, and valuable-metal recovery are prioritized. It can achieve bulk arsenic removal directly under acidic conditions and enable selective separation of components such as Cu and As through staged process control [1,6,11,20,23]. Although lime neutralization and lime-ferric co-precipitation consume acidity and generate relatively large amounts of sludge, their process maturity and operational stability make them important for residual-acid neutralization and subsequent advanced treatment [1,59,70,76,79]. In particular, the HDS process can increase sludge solids content, improve settling performance, and reduce sludge volume through sludge recycling and seeding effects [71,72]. When long-term residue stability is the primary objective, scorodite precipitation is more attractive, although it requires tighter control of pH, temperature, oxidation, and crystallization conditions [78,83,86,87,88,89,90]. Adsorption, membrane separation, and ion exchange are more suitable for advanced treatment or final polishing after bulk contaminant removal [15,92,93,94].

Accordingly, practical process selection should be based on influent acidity, As concentration and oxidation state, coexisting metals, resource recovery requirements, residue management constraints, and existing plant infrastructure, rather than solely on aqueous-phase As removal efficiency. For high-arsenic HAAW, a representative integrated route is “selective arsenic removal/valuable-metal recovery by sulfide precipitation + lime neutralization or lime-ferric treatment for residual-acid neutralization and advanced purification” [1,6,20,23]. Where long-term arsenic immobilization is required, scorodite precipitation may also be considered as an alternative stabilization route for suitable As(V)-bearing streams [78,83,84,85]. Overall, HAAW treatment should be evaluated as an integrated system linking aqueous-phase purification, resource recovery, and arsenic-bearing residue management. The following section therefore focuses on sulfide precipitation, which is one of the major treatment routes for high-concentration acidic HAAW and remains a key area of current research [1,6,20,23].

## 4. Sulfide Precipitation: Thermodynamic and Process Fundamentals

Sulfide precipitation has become one of the major technologies for arsenic removal from HAAW. Compared with lime-based processes, which generally require substantial pre-neutralization, sulfide precipitation can be conducted directly under the strongly acidic conditions of HAAW, removing arsenic and coexisting heavy metals as sparingly soluble sulfides [1,5,6,20]. Figure 5 presents a general process flow for sulfide precipitation treatment of HAAW. Following solid–liquid separation, an arsenic-rich sulfide residue and an acidic filtrate are generated; depending on the wastewater composition and treatment objectives, the acidic filtrate may undergo a second-stage sulfide polishing step before subsequent treatment or reuse. This section systematically discusses sulfide precipitation in terms of its thermodynamic basis, sulfide-reagent characteristics, selective separation, and process integration.

### 4.1. Thermodynamic Foundations

#### 4.1.1. As-S-H_2_O System

The thermodynamic behavior of arsenic in sulfide-bearing systems is elegantly captured by the E-pH (Pourbaix) diagram for the As-S-H_2_O system, shown in Figure 6. When sulfide species are introduced into an arsenic-bearing solution, a broad stability region for solid As_2_S_3_ can appear under appropriate thermodynamic conditions. Under the thermodynamic assumptions and activity conditions adopted for Figure 6, As_2_S_3_ is thermodynamically favored over a broad region; in the calculated diagram, this includes strongly acidic conditions (pH < 2) and reducing to mildly oxidizing potentials (E < 0.5 V) [5,20]. This thermodynamic prediction is consistent with experimental observations: in high-concentration H_2_SO_4_ systems, H_2_S precipitation achieves arsenic removal efficiencies of 99.87%, reducing residual arsenic to 0.28 mg/L [97].

As_2_S_3_ exhibits low solubility in acidic media, providing an important thermodynamic basis for arsenic removal by sulfide precipitation. Under strongly acidic conditions, dissolved arsenic can be separated from the aqueous phase through the formation of sparingly soluble As_2_S_3_. In general, metal sulfides typically exhibit lower solubilities than their corresponding metal hydroxides, which constitutes an important thermodynamic advantage of sulfide precipitation for the removal of dissolved metals [20,98]. In a systematic review of metal sulfide precipitation, Lewis [98] demonstrated that substantial differences exist in the dissolution behavior and thermodynamic stability of individual metal sulfides, while solution pH can further influence their precipitation equilibria by regulating the distribution of aqueous sulfide species.

As shown in Figure 7, the distribution of aqueous sulfide species exhibits a pronounced pH dependence under the adopted acid-base dissociation equilibrium parameters [6,98]. The figure is intended to illustrate the qualitative pH-dependent speciation trend under the adopted acid-base equilibrium parameters rather than to serve as a quantitative model for high-ionic-strength HAAW. Under acidic conditions, dissolved sulfide exists predominantly as H_2_S(aq); as pH increases, HS^−^ gradually becomes the dominant species, whereas S^2−^ attains an appreciable relative abundance only under strongly alkaline conditions. Variations in sulfide speciation affect the effective sulfide activity and precipitation equilibria within the system. Meanwhile, the predominance of H_2_S(aq) under strongly acidic conditions also increases the potential for H_2_S formation and volatilization. Therefore, pH is a key parameter governing both the thermodynamic behavior and operational safety of sulfide precipitation [6,20,98].

For HAAW, the relatively low solubility of As_2_S_3_ under strongly acidic conditions enables effective arsenic removal by sulfide precipitation while largely preserving the acidity of the solution, thereby reducing the neutralizing-agent consumption and acidity loss associated with the substantial pH increase required in conventional lime-based treatment [1,5,20,97]. This characteristic makes sulfide precipitation particularly suitable for highly acidic HAAW systems in which acidity may fluctuate with feed composition and upstream operating conditions. Nevertheless, the dissolution and stability of As_2_S_3_ are not governed solely by pH but are jointly affected by temperature, redox conditions, sulfide concentration, ionic strength, and coexisting species [5,6,18,20]. Therefore, its low solubility should not be interpreted as complete insensitivity to variations in pH. In addition, the low solubility of metal sulfides and their rapid precipitation kinetics impose stringent requirements on sulfide dosage and process control. Insufficient sulfide addition may result in incomplete precipitation of target species, whereas excessive addition may lead to the accumulation of residual sulfide and increase the safety and environmental risks associated with H_2_S formation and volatilization [6,20,98].

#### 4.1.2. Stoichiometry of Arsenic Sulfidation

The stoichiometric requirements for arsenic sulfidation depend critically on arsenic valence state. For As(III), the precipitation reaction is [18]:

2H_3_AsO_3_ (aq) + 3H_2_S (aq) → As_2_S_3_ (s) + 6H_2_O (l) (9)

This reaction corresponds to a theoretical S/As molar ratio of 1.5.

For As(V), however, the situation is fundamentally different. As(V) sulfidation involves reduction and sulfur-containing intermediates before the formation of As_2_S_3_ [18,99]. The overall stoichiometry can be represented as [18]:

2H_3_AsO_4_ (aq) + 5H_2_S (aq) → As_2_S_3_ (s) + 2S (s) + 8H_2_O (l) (10)

This reaction has a theoretical S/As molar ratio of 2.5—substantially higher than for As(III). Importantly, arsenate reduction by dissolved sulfide can proceed rapidly under moderately acidic conditions, whereas dissolved arsenic–sulfide intermediates may persist for considerably longer periods; the formation and transformation of these intermediates can therefore become important kinetic limitations in the overall As(V) sulfidation pathway [18,99]. Thus, the overall As(V) sulfidation pathway is kinetically more complex than that of As(III) [18,99]. In real HAAW, coexisting species further affect sulfide consumption and overall sulfidation kinetics [7,11,18]. This valence-state dependence of stoichiometry is one of the primary reasons why sulfide reagent dosing in industrial practice is so challenging: HAAW typically contains both As(III) and As(V) in proportions that vary with feed composition and upstream process conditions [1,5,6,18].

#### 4.1.3. Competitive Precipitation of Metal Sulfides

HAAW is not a single-solute system. In addition to arsenic, the wastewater contains Cu^2+^, Pb^2+^, Zn^2+^, Cd^2+^, and other metal cations that also react with sulfide to form sparingly soluble precipitates. The thermodynamic tendency for precipitation is related to the ion activity product and the solubility product (K_sp_) of the corresponding sulfide. Representative reported values at approximately 25 °C are shown in Table 6:

The reported K_sp_ values span many orders of magnitude, providing an important thermodynamic basis for selective sulfide precipitation. Nevertheless, a universal precipitation sequence cannot be inferred directly from individual K_sp_ values because metal sulfides differ in stoichiometry, and their conditional solubilities are strongly affected by pH, sulfide speciation, complexation, and solution composition [5,6,20,98]. This limitation is particularly relevant to HAAW, where multiple metal ions, high ionic strength, sulfate and chloride complexation, and kinetic and mass-transfer effects can substantially alter the ideal thermodynamic selectivity. Consequently, selective precipitation in practical systems depends not only on intrinsic solubility equilibria but also on the controlled regulation of sulfide activity, pH, solution potential, and species distribution [1,5,6,20,98]. These coupled effects constitute an important part of the theoretical challenges discussed in Section 6.

### 4.2. Sulfide Reagents and Their Characteristics

The type and delivery mode of sulfide reagents directly affect effective sulfur utilization, local supersaturation, particle formation, and H_2_S-related safety risks during sulfide precipitation of HAAW [6,13,20]. Commonly used or extensively investigated sulfur sources include soluble sulfide reagents such as Na_2_S/NaHS, gaseous H_2_S, and sparingly soluble controlled-release sulfur sources such as FeS/ZnS [1,5,6,16,17,20,98]. These sulfur sources differ markedly in sulfur-delivery mode, mass-transfer pathway, reaction kinetics, and engineering safety.

#### 4.2.1. Soluble Sulfide Reagents: Na_2_S and NaHS

Na_2_S and NaHS are commonly used soluble sulfide reagents for HAAW treatment because of their wide availability, relatively low cost, rapid dissolution, and ease of operation [5,6,20,98]. After dissolution, Na_2_S rapidly dissociates and undergoes stepwise protonation in strongly acidic media:

Na_2_S (aq) → 2Na^+^ (aq) + S^2−^ (aq) (11)

S^2−^ (aq) + H^+^ (aq) ⇌ HS^−^ (aq) (12)

HS^−^ (aq) + H^+^ (aq) ⇌ H_2_S (aq) (13)

H_2_S (aq) ⇌ H_2_S (g) (14)

The distribution of these sulfide species is governed primarily by pH and is also affected by factors such as temperature and ionic strength. In strongly acidic HAAW at pH < 2, dissolved S(−II) exists predominantly as H_2_S(aq). When the system is insufficiently sealed, or when agitation or aeration enhances gas–liquid mass transfer, dissolved H_2_S can further escape from the liquid phase to the gas phase [6,20,98]. Rapid protonation and H_2_S volatilization reduce effective sulfur utilization. Consequently, the practical sulfide demand may exceed the theoretical stoichiometric requirement and must be optimized for the specific wastewater composition and reactor configuration [1,5,6,20,98]. Escaping H_2_S also poses a significant occupational health risk, with an immediately dangerous to life or health (IDLH) concentration of approximately 100 ppm; exposure to elevated concentrations can cause severe acute health effects and may be fatal [100]. In addition, Na^+^ introduced by Na_2_S/NaHS remains in the aqueous phase, increasing the salt load of the treated wastewater and potentially complicating subsequent treatment and reuse [1,5,6].

In addition to sulfide protonation and H_2_S volatilization, sulfide dosing and mixing scales are important factors affecting treatment performance. Because sulfide precipitation is rapid, locally high sulfide concentrations near the reagent addition point can generate high supersaturation before complete mixing, favoring H_2_S loss and the formation of fine sulfide particles [6,13,101]. This indicates that the average sulfide concentration in the reactor does not necessarily represent the local reaction environment that governs sulfur utilization, nucleation behavior, and final particle characteristics.

Li et al. [102] investigated single-stage sulfide removal of arsenic from high-arsenic smelting waste acid with an initial As concentration of 16,060 mg/L using Na_2_S·9H_2_O as the sulfide reagent. The study demonstrated that sulfide dosage, dosing mode, reaction time, temperature, and subsequent flocculation affected treatment performance. Under the optimized process, the residual As concentration was reduced to 0.23 mg/L and could be further decreased to <0.1 mg/L, while Pb and Cu were both reduced to <0.1 mg/L [102]. These results demonstrate that coordinated control of sulfide dosage, dosing strategy, and reaction time is important for achieving efficient arsenic removal.

#### 4.2.2. Gaseous Sulfide Reagent: H_2_S

Compared with soluble sulfide reagents such as Na_2_S and NaHS, direct H_2_S injection offers the main advantage of avoiding additional Na^+^ input while enabling continuous sulfur supply through regulation of the gas flow rate [10,20,97]. Its treatment performance depends not only on the sulfide precipitation reaction but also on gas holdup, bubble size, gas–liquid interfacial area, liquid-phase mixing, pressure, and the consumption rate of dissolved H_2_S [10,13,20]. Therefore, even when the precipitation reaction between H_2_S and dissolved arsenic is intrinsically rapid, gas–liquid mass transfer may still become an important factor affecting the overall reaction rate under specific operating conditions. This issue is discussed further in Section 7.1.

Engineering applications have demonstrated the feasibility of H_2_S sulfidation for HAAW treatment. At a treatment scale of 1920 m^3^/d, wastewater containing approximately 4500 mg/L As was treated to a stable effluent concentration below 10 mg/L, corresponding to an arsenic removal efficiency of approximately 99.8% [103]. In a high-H_2_SO_4_ system, an arsenic removal efficiency of 99.87% and a residual arsenic concentration of 0.28 mg/L were achieved under optimized H_2_S supply conditions [97]. Because no Na^+^ is introduced, H_2_S can reduce the salt load of the treatment system and potentially alleviate the desalination burden associated with subsequent treatment and wastewater reuse.

However, H_2_S is acutely toxic, and its industrial application requires dedicated gas generation or storage facilities, corrosion-resistant transport and injection systems, as well as H_2_S online monitoring, interlock control, and off-gas treatment systems [10,20,100]. Therefore, evaluations of the economics and engineering scalability of H_2_S relative to soluble sulfide reagents should consider gas–liquid mass-transfer efficiency, safety infrastructure, and operation and maintenance costs.

#### 4.2.3. Controlled-Release Sulfide Sources: FeS and ZnS

Sparingly soluble metal sulfides such as FeS and ZnS can serve as controlled-release sulfur sources by gradually dissolving in acidic media and releasing dissolved S(−II) species, which exist predominantly as H_2_S(aq) under strongly acidic conditions [6,16,17,20]. Compared with Na_2_S/NaHS, their slower sulfur release can moderate transient sulfide concentrations and may reduce local supersaturation and rapid H_2_S generation, thereby potentially improving the matching between sulfur supply and sulfide consumption by arsenic and coexisting metals [6,16,17].

The dissolution and sulfur release of FeS and ZnS are typical fluid-particle heterogeneous processes. Their apparent rates may be influenced by external liquid-film mass transfer and surface reaction, while diffusion through product or passivation layers may also contribute to the overall resistance under specific conditions. Therefore, crystal structure, specific surface area, and surface properties can significantly affect sulfur-release kinetics [16,17]. These heterogeneous reaction mechanisms and potential rate-controlling steps are further discussed in Section 7.1.

Among these sulfur sources, FeS has been extensively investigated. Its simplified overall dissolution reaction in acidic solution can be expressed as follows [16,20]:

FeS (s) + 2H^+^ (aq) → Fe^2+^ (aq) + H_2_S (aq) (15)

The released H_2_S subsequently participates in arsenic sulfide precipitation. Chen et al. [16] applied high-temperature-synthesized monoclinic FeS to HAAW treatment. At an S/As molar ratio of 3.75, 40 °C, and 150 min, the As concentration decreased from 1889 mg/L to 1.54 mg/L, corresponding to a removal efficiency of 99.92% [16]. Solid-phase characterization indicated that As(III) was removed primarily as As_2_S_3_, while the FeS surface may also provide sites for heterogeneous nucleation and deposition of As_2_S_3_ [16]. Fe^2+^ released during FeS dissolution can also be oxidized to Fe(III) under subsequent aeration or other oxidative conditions. As the pH increases, Fe(III) hydrolyzes to form iron (oxyhydr)oxides, which can further remove residual arsenic through adsorption or co-precipitation [73,74]. In staged FeS−lime treatment, an FeS dosage twice the theoretical requirement decreased the As concentration from 6240 mg/L to <0.5 mg/L, with an average removal efficiency exceeding 99.9% [104]. Thus, FeS can function both as a controlled-release sulfur source and as a component of an integrated treatment process combining sulfide precipitation with subsequent neutralization and iron (oxyhydr)oxide-mediated purification.

ZnS has also been investigated as a controlled-release sulfur source for arsenic removal [17]. Under optimized ultrasound-assisted conditions of an acidity of 120 g/L, an S/As molar ratio of 3:1, 60 °C, an ultrasonic power of 100 W, and a reaction time of 120 min, the arsenic removal efficiency reached 99.96%, with a residual As concentration of 0.42 mg/L [17]. Ultrasonic treatment can further promote ZnS dissolution and arsenic sulfide precipitation by improving particle dispersion, interfacial mass transfer, and surface renewal [17].

Overall, Na_2_S/NaHS provide rapid reaction and operational simplicity but may cause local supersaturation, Na^+^ introduction, and H_2_S release. Gaseous H_2_S enables controllable sulfur supply without increasing the Na^+^ load but requires effective gas–liquid mass transfer and stringent safety control. In contrast, FeS and ZnS provide more gradual sulfur release and may promote heterogeneous nucleation, but generally require longer reaction times and may be constrained by heterogeneous mass transfer, surface reaction, passivation, and the introduction of Fe or Zn [6,16,17,20]. Representative performances and engineering characteristics of these sulfur sources are summarized in Table 7.

### 4.3. Selective Sulfide Separation

The thermodynamic differences in metal sulfide solubilities provide the basis for selective separation of metals from HAAW. By controlling sulfide activity and, where applicable, the solution potential (Eh or ORP), different metals can be precipitated sequentially [6,98,105,106,107,108].

#### 4.3.1. ORP-Controlled Selective Precipitation

The principle of ORP-controlled selective precipitation is that different sulfide precipitation reactions can be favored over different potential ranges [6,98,106,107]. Owing to its extremely low solubility, copper sulfide can precipitate preferentially at relatively high potentials. In contrast, the precipitation behavior of As- and Zn-bearing sulfides is more sensitive to the specific solution chemistry, and their effective potential windows vary with system conditions [6,98,106,107]. Therefore, a universal precipitation sequence for these sulfides cannot be established solely on the basis of K_sp_ values.

Laboratory and production studies have demonstrated the effectiveness of ORP control [105,109]. Using H_2_S as the sulfide reagent, controlling the first-stage ORP at 245 mV selectively precipitates copper, while reducing the ORP to 10 mV in the second stage precipitates arsenic [109]. Figure 8 illustrates the two-stage selective sulfide separation process. The results are striking: after two-stage treatment, copper and arsenic concentrations in the wastewater are reduced to 0.03 mg/L and 0.22 mg/L, respectively [109]. These results demonstrate that staged ORP control can achieve deep Cu/As removal while separating the two precipitation steps [109].

#### 4.3.2. Resource Recovery Implications

The significance of selective sulfide separation extends beyond pollution control to resource recovery. Traditional co-precipitation approaches that remove all metals together produce mixed Cu–As-containing hazardous residues that are difficult to recover selectively [1,5,105]. Selective separation, by contrast, generates a Cu-enriched sulfide residue that can be returned to the smelting circuit under suitable process conditions [105]. Arsenic is concentrated in a separate stream, facilitating its stabilization or further processing [1,5,105].

This shift from “treatment for disposal” to “treatment for recovery” aligns with the broader industry trend toward circular economy principles and increasing emphasis on resource recovery and waste minimization [1,12,110].

### 4.4. Process Integration: Sulfide + Subsequent Treatment

As shown in Figure 5, the downstream treatment route following sulfide precipitation depends on the specific treatment objective and may include subsequent neutralization, acid recovery, or reuse. Although sulfide precipitation can effectively remove arsenic and heavy metals, it does not eliminate the residual acidity of the wastewater [1,5]. For discharge-oriented treatment, further neutralization is generally required; where reuse is feasible, the acidic filtrate may instead be further purified to enable acid recovery or reuse [1,12,110]. In industrial practice, sulfide precipitation is therefore commonly followed by lime neutralization or lime–iron salt treatment [1,5,110]. As a typical integrated treatment configuration, Figure 9 illustrates the combined “sulfide precipitation + lime neutralization” process, which is one of the commonly applied treatment routes for HAAW in the non-ferrous smelting industry [1,5,110].

The “sulfide + lime” combination is a common industrial treatment train for HAAW [1,5,110]. Sulfide precipitation removes the bulk of arsenic and heavy metals selectively, producing a relatively small volume of As-rich sulfide sludge [1,5]. The sulfide-treated effluent, now with low contaminant levels but still acidic, then undergoes lime neutralization to raise pH to discharge or reuse standards [110,111]. Because the bulk of the arsenic has already been removed, the arsenic load entering the subsequent neutralization stage is reduced [1,5,110].

The “sulfide + scorodite” combination represents a more advanced integration. Sulfide precipitation concentrates arsenic into a small-volume As_2_S_3_ stream; this stream can then be oxidized and converted to scorodite under controlled hydrothermal conditions, with approximately 99% As immobilization reported [112]. This approach combines the efficiency of sulfide precipitation with improved residue stability, though it requires more sophisticated process control.

### 4.5. Interim Assessment of Sulfide Precipitation

Overall, sulfide precipitation offers distinct process advantages for treating high-concentration, strongly acidic HAAW. Under suitable conditions, the process can operate directly at low pH without pre-neutralization while achieving high arsenic removal efficiencies [1,5,20,97]. The resulting arsenic-rich sulfide residues are relatively low in volume, and coexisting metals such as Cu, Pb, and Cd can be removed simultaneously [1,5,6,20]. By exploiting differences in the precipitation behavior of metal sulfides and regulating pH, redox conditions, and sulfide supply, selective separation of arsenic from valuable metals can also be achieved, thereby facilitating subsequent resource recovery [6,11,12,105,106,107,108,109]. Therefore, compared with treatment routes dominated by neutralization, sulfide precipitation offers potential advantages in acidity preservation, sludge minimization, and selective separation of valuable metals [1,5,6,20].

However, its practical engineering performance is constrained by multiple factors. Variations in the As(III)/As(V) ratio in HAAW, competitive reactions with coexisting metals, and sulfur losses caused by H_2_S volatilization and sulfide oxidation make it difficult to determine the actual sulfide demand accurately from theoretical stoichiometry alone [7,11,18,20]. Under strongly acidic conditions, rapid protonation of sulfide further increases H_2_S release and associated safety-control requirements [6,20,98]. Meanwhile, rapid reaction kinetics and high local supersaturation can induce extensive nucleation of fine As_2_S_3_ particles and may promote the formation of stable colloids, thereby complicating subsequent flocculation, settling, and filtration [13,14,101]. The use of Na_2_S or NaHS also introduces Na^+^ into the aqueous phase, increasing the salt load [1,5,6]. In addition, arsenic sulfide residues are generally amorphous or poorly crystalline, and their long-term oxidation, dissolution, and phase-transformation behavior remains uncertain [8,9,112,113].

Therefore, the technical advantages of sulfide precipitation cannot be fully realized in all HAAW systems. Its actual performance depends on influent composition, sulfide type and delivery mode, reactor mixing and mass-transfer conditions, H_2_S control, solid–liquid separation, and subsequent residue stabilization [1,5,6,10,13,20]. Sulfide precipitation should therefore be selected according to specific wastewater characteristics and engineering objectives rather than regarded as a universally optimal treatment technology. The next section further analyzes the major engineering bottlenecks associated with sulfide precipitation, while Section 6 discusses the fundamental knowledge gaps underlying these engineering challenges from the perspectives of thermodynamics, nucleation and growth, and interfacial chemistry.

## 5. Engineering Bottlenecks of Sulfide Precipitation

Although sulfide precipitation provides favorable thermodynamic driving forces under suitable conditions and has established a certain engineering application basis, its stable operation in HAAW treatment remains constrained by four interrelated engineering bottlenecks: difficulty in accurately determining sulfide dosage, low sulfide utilization efficiency and H_2_S control, formation of fine As_2_S_3_-rich particles and associated solid–liquid separation difficulties, and uncertainty in the long-term stability of arsenic sulfide residues. These issues are not independent of one another. The sulfide supply mode affects the local effective sulfur concentration and supersaturation; supersaturation further controls particle nucleation and growth; particle properties determine subsequent aggregation and solid–liquid separation behavior; and the composition and structure of the final solids influence the long-term stability of the residues. Therefore, the engineering challenges of sulfide precipitation for HAAW essentially involve multiscale coupling among sulfur supply, mass transfer and reaction, particle formation, solid–liquid separation, and solid-phase evolution, which also provides the basis for the mechanistic analyses presented in Section 6 and Section 7.

### 5.1. Difficulty in Accurately Determining Sulfide Dosage

Sulfide dosage directly affects arsenic removal efficiency, reagent consumption, residual sulfide concentration, and H_2_S-related safety risks, yet the theoretical requirement is difficult to translate directly into a stable and reliable industrial set point. First, the baseline sulfide demand depends on the oxidation state of arsenic. As discussed in Section 4.1.2, As(III) and As(V) differ markedly in their sulfidation pathways and effective sulfur requirements, while arsenic speciation in metallurgical wastewater varies with wastewater composition and redox history [7,18,99,114]. Therefore, total arsenic concentration alone is insufficient to accurately determine the theoretical sulfide requirement.

Meanwhile, coexisting metals such as Cu, Pb, Zn, and Cd also react with sulfide and create additional competitive sulfur demand [6,7,11,98]. Differences in the solubility, precipitation onset conditions, and reaction rates of individual metal sulfides cause the instantaneous sulfur demand to vary continuously during the precipitation process. Therefore, sulfide demand depends not only on arsenic concentration and oxidation state, but also on the composition of coexisting metals and their competitive precipitation behavior.

Another source of uncertainty is ineffective sulfur loss. Under strongly acidic conditions, sulfide is rapidly protonated to H_2_S, which can subsequently escape from the liquid phase to the gas phase [10,101]. In oxidizing matrices, part of S(−II) can also be converted to S(0) or other sulfur-containing species [18,114]. These losses are jointly affected by reagent addition location, mixing conditions, gas–liquid mass transfer, and the redox environment [10,101,114]. Consequently, empirical excess-dosage factors cannot be assumed to remain constant across different plants, influent compositions, or operating conditions. Thus, determination of sulfide dosage is intrinsically governed by the combined effects of stoichiometry, competitive reactions, and non-target sulfur losses.

### 5.2. Low Sulfide Utilization Efficiency and H_2_S Control

Low sulfide utilization efficiency is closely related to the difficulty in accurately determining sulfide dosage. Not all sulfur introduced into the reaction system participates in the formation of target arsenic sulfides, and its actual utilization depends on the relative rates of sulfur-source supply, mass transfer, precipitation reactions, and non-target losses.

For soluble sulfide reagents such as Na_2_S and NaHS, sulfide is rapidly protonated in strongly acidic media, and relatively high concentrations of dissolved H_2_S may form near the dosing point before being fully consumed by precipitation reactions. When local mixing is insufficient or gas–liquid interfacial mass transfer promotes H_2_S release, transfer of H_2_S from the liquid to the gas phase competes with the target precipitation reaction, thereby reducing effective sulfur utilization [10,98,101]. Systems supplied directly with gaseous H_2_S are subject to a different limiting mechanism: when gas–liquid mass-transfer capacity is insufficient, H_2_S transfer from the gas phase may not match its consumption by precipitation in the liquid phase, making mass transfer an important factor affecting the overall reaction [10,101]. For controlled-release sulfur sources such as FeS and ZnS, sulfur release may instead be limited by particle dissolution, surface reaction and, under specific conditions, diffusion and surface passivation [16,17,114].

Therefore, sulfide utilization does not have a single rate-controlling step applicable to all sulfur sources and reactor configurations, but rather depends on the relative timescales of supply, mass transfer, reaction, and loss processes. Although excess sulfide addition can reduce the risk of effluent non-compliance caused by underdosing, it increases the risks associated with H_2_S exposure, off-gas treatment load, residual sulfide, and formation of secondary sulfur phases. Conversely, overly conservative dosing may result in residual dissolved arsenic or valuable metals. Industrial process control should therefore not rely solely on a fixed multiple of the theoretical S/As ratio, but should, where possible, be dynamically adjusted according to the effective sulfur-supply state and actual reaction progress [10,11,101].

### 5.3. Formation of Fine As_2_S_3_-Rich Particles and Difficulties in Solid–Liquid Separation

Another major engineering bottleneck in arsenic removal by sulfide precipitation is the formation of fine As_2_S_3_-rich particles and the resulting difficulty in solid–liquid separation. Sulfide precipitation reactions are generally rapid. When sulfide dosing and mixing rates cannot match the reaction rate, local supersaturation near the dosing point can become much higher than the average level in the bulk solution, thereby promoting extensive nucleation of fine particles [13,14,101,115]. These newly formed particles may further develop into stable suspensions, meaning that conversion of dissolved arsenic into a solid phase does not necessarily guarantee efficient settling or filtration [14,115].

It should be noted that whether particles form stable colloids depends not only on their nominal size, but also on particle surface composition, surface charge, ionic strength, adsorbed sulfide species, co-precipitated metal sulfides, and subsequent aging, seeding, and coagulation processes [14,115]. Therefore, the favorable settling or dewatering performance reported for general mixed-metal sulfide sludges cannot be directly extrapolated to newly formed fine As_2_S_3_-rich particles.

#### 5.3.1. Mechanism of Fine-Particle Formation

When soluble sulfide reagents are introduced into acidic arsenic-containing solutions, rapid precipitation can generate high local supersaturation before complete mixing is achieved in the dosing zone [13,101]. The resulting locally high supersaturation markedly increases the nucleation rate, causing the system to favor the formation of large numbers of new particles rather than continued deposition of solutes onto existing solid surfaces.

According to classical homogeneous nucleation theory, a commonly used simplified expression for the nucleation rate is [116]:

(16) $$J=\mathrm{A exp}[-\frac{B}{{\left(\ln S\right)}^{2}}]$$

where J is the homogeneous nucleation rate, S is the supersaturation ratio, A is a kinetic pre-exponential factor related to molecular transport and particle attachment, and B is a parameter associated with interfacial energy, temperature, and the nucleation free-energy barrier. This expression is used here primarily to illustrate the high sensitivity of nucleation rate to supersaturation rather than as a direct quantitative model for predicting As_2_S_3_ nucleation rates in complex HAAW systems. High local supersaturation favors the simultaneous formation of numerous new nuclei, whereas lower and controlled supersaturation, together with a larger available surface area of pre-existing solids, is more favorable for solute deposition onto existing particles and subsequent particle growth [13,14,116]. Therefore, sulfide addition location, dosing rate, and reactor micromixing conditions directly affect the final particle number and particle-size distribution. Figure 10 schematically illustrates the formation of fine As_2_S_3_ particles under high-supersaturation conditions and their subsequent colloidal stabilization.

#### 5.3.2. Colloidal Stabilization Mechanism

In addition to particle size, interfacial charge and interparticle interactions are important factors determining whether As_2_S_3_-rich particles remain stably suspended over extended periods. Fine As_2_S_3_ particles can exhibit substantial colloidal stability under sulfide-precipitation conditions [14,115]. In classical colloid chemistry, H_2_S-stabilized As_2_S_3_ sols are commonly represented schematically by adsorption of HS^−^; the following micellar expression is retained here as a conceptual model rather than a directly resolved surface structure of As_2_S_3_ in HAAW:

[(As_2_S_3_)m·nHS^−^·(n − x)H^+^]^x−^·xH^+^ (17)

Interactions between charged particles are interpreted here within the extended DLVO (XDLVO) framework, in which Lifshitz-van der Waals and electrostatic double-layer interactions are supplemented by Lewis acid-base interactions [117]. When the total interaction-energy profile exhibits a sufficiently high repulsive barrier, particles are unable to approach and aggregate readily, thereby exhibiting relatively high kinetic stability. Solution ionic strength and counterion valence can further affect particle aggregation by altering the electrical double-layer structure and the interaction-energy barrier [117,118].

However, actual HAAW differs substantially from ideal dilute-electrolyte systems. Its relatively high ionic strength compresses the electrical double layer, while specific adsorption, multivalent ions, co-precipitated sulfides, iron or aluminum hydrolysis products, and surface heterogeneity may further alter particle surface potential and interparticle interactions. Accordingly, even XDLVO should be regarded primarily as a mechanistic framework rather than a stand-alone quantitative predictor for complex HAAW matrices [117,118]. Therefore, As_2_S_3_ colloidal stability should, wherever possible, be evaluated using ζ-potential, particle-size distribution, and aggregation behavior measured in actual HAAW matrices rather than inferred solely from general relationships derived for ideal electrolyte systems. More specific interfacial-chemistry relationships, including the critical coagulation concentration and the Schulze-Hardy rule, are discussed further in Section 6.3, where the corresponding particle-interaction framework is examined.

#### 5.3.3. Engineering Implications and Control Strategies

The formation of As_2_S_3_-rich colloids can prolong settling time, reduce filtration rates, and increase the load on downstream solid–liquid separation equipment. Fe(III)- and Al(III)-based coagulants can promote destabilization and coagulation of fine As_2_S_3_ particles [119], whereas PAM can promote the formation of larger flocs and improve settling performance [14]. However, enhanced coagulation-flocculation may also increase reagent consumption and sludge production and introduce additional ions or iron/aluminum hydrolysis products, thereby lowering the arsenic grade of the residue and increasing the complexity of subsequent stabilization or resource-recovery treatment.

Beyond downstream coagulation-flocculation, a more targeted strategy is to regulate particle formation during the precipitation stage. For example, reducing the local sulfide concentration, optimizing reagent addition location and dosing mode, improving micromixing, or introducing seed particles and controlled-release sulfur sources such as FeS/ZnS can moderate local supersaturation and favor particle growth or aggregation, thereby alleviating the burden on subsequent solid–liquid separation [13,14,16,17,101,119]. The common objective of these measures is to avoid reaction rates that greatly exceed the rates of mixing and particle growth, thereby achieving better matching between sulfide supply and particle formation.

Therefore, the engineering objective of sulfide precipitation should not be limited to maximizing the instantaneous chemical reaction rate, but should instead focus on regulating the spatiotemporal supersaturation history within the reactor so that arsenic conversion, particle nucleation and growth, particle aggregation, and final dewatering are mutually compatible. Fundamentally, this issue involves solution chemistry, particle population dynamics, and reactor mixing.

### 5.4. Long-Term Stability of Arsenic Sulfide Residues

Sulfide precipitation can efficiently transfer dissolved arsenic into the solid phase, but this transformation does not necessarily ensure long-term arsenic stabilization. In sulfide precipitation systems dominated by As(III), newly formed arsenic-bearing solids commonly contain amorphous or poorly crystalline As_2_S_3_-rich phases as important arsenic-bearing components and may also contain co-precipitated metal sulfides, elemental sulfur, and other solid phases [8,18]. The environmental behavior of amorphous As_2_S_3_ can differ substantially from that of crystalline arsenic sulfides, and crystallinity is an important factor affecting arsenic and sulfur release [120,121]. The phase composition, crystallinity, particle size, pore structure, and coexisting phases of the residues may all affect their subsequent environmental behavior. Therefore, evaluation of sulfide-precipitation residues should not focus solely on the initial arsenic removal efficiency, but should also consider their solid-phase structure and long-term evolution characteristics [8,120,121].

During long-term storage, transportation, and disposal, arsenic sulfides may undergo oxidation, dissolution, or phase transformation under the influence of pH, redox conditions, dissolved oxygen, temperature, and coexisting ions [8,120,121]. In particular, under oxidizing conditions, both As(III) and S(−II) in As_2_S_3_ may be oxidized, leading to transformation of the original sparingly soluble sulfide solid phase and potentially promoting the formation and release of soluble arsenic species. The simplified limiting reaction for complete oxidation of As_2_S_3_ can be expressed as [120,121]:

As_2_S_3_ (s) + 7O_2_ (g) + 6H_2_O (l) → 2H_3_AsO_4_ (aq) + 3SO_4_^2−^ (aq) + 6H^+^ (aq) (18)

This equation represents the overall stoichiometric relationship under complete oxidation conditions, whereas the actual oxidation of arsenic sulfide residues may involve more complex processes, including the formation of intermediate-valence sulfur species, localized dissolution, and secondary solid-phase formation [120,121]. Released arsenic may also be re-immobilized through secondary adsorption or precipitation; therefore, its ultimate mobility depends on the competition among arsenic sulfide oxidation, dissolution, and secondary immobilization processes. Accordingly, evaluating process environmental safety solely on the basis of aqueous-phase arsenic removal during sulfide precipitation is insufficient. Residue stability should also be comprehensively assessed in terms of solid-phase composition, crystallinity, long-term leaching behavior, and aging processes [8,120,121].

Solid-phase characterization is essential for identifying residue structure and its long-term evolution. X-ray diffraction (XRD) can be used to identify crystalline arsenic sulfides and coexisting crystalline phases and to track phase transformations occurring during aging or stabilization. However, newly formed As_2_S_3_ may be amorphous or poorly crystalline, resulting in weak diffraction peaks that may be difficult to resolve by conventional XRD [8,18]. Therefore, the absence of distinct diffraction peaks does not directly demonstrate the absence of arsenic sulfides. For such complex residues, XRD should preferably be combined with solid-phase characterization techniques such as XPS, Raman spectroscopy, and SEM-EDS/TEM, together with leaching tests [8,18,112,113]. Comparative characterization before and after aging or leaching can then be used to establish the relationship of process conditions → solid-phase structure → residue stability → arsenic release.

In addition, upstream sulfidation conditions can influence the final properties of the residues. Particle size and crystallinity established during precipitation can affect subsequent interfacial reactivity and arsenic release [8,13,120,121], while co-precipitated metal sulfides, hydrolysis products of coagulants, and subsequent stabilization materials may further alter arsenic speciation and reactive interfaces. Therefore, the long-term stability of the residues is not an end-of-pipe issue independent of upstream reactions, but rather the combined outcome of sulfur-source selection, local supersaturation, particle formation, co-precipitation behavior, solid–liquid separation, and subsequent aging.

Overall, the four engineering bottlenecks of sulfide precipitation form a continuous causal chain: uncertainties in sulfide demand and supply affect effective sulfur utilization and local supersaturation; local supersaturation controls particle nucleation and growth; particle properties further determine solid–liquid separation performance; and the composition and structure of the resulting solids ultimately influence the long-term stability of the residues. Therefore, stable scale-up of sulfide precipitation for HAAW cannot be achieved by optimizing a single operating parameter alone. Instead, the intrinsic relationships among these bottlenecks must be understood across multiple scales, including thermodynamics, nucleation and growth, interfacial chemistry, and reaction engineering. Section 6 further discusses the underlying fundamental knowledge gaps, whereas Section 7 analyzes the corresponding engineering control mechanisms from the perspective of coupled reaction, mass transfer, and mixing.

## 6. Fundamental Knowledge Gaps

The four engineering bottlenecks discussed in Section 5—difficulty in accurately determining sulfide dosage, low sulfide utilization efficiency and H_2_S control, formation of fine As_2_S_3_-rich particles and associated solid–liquid separation difficulties, and uncertainty in the long-term stability of arsenic sulfide residues—largely arise from the lack of quantitative understanding of species equilibria, sulfur supply and mass transfer, particle formation, and interfacial interactions in complex HAAW systems.

From the perspective of the overall sulfide precipitation process, the current fundamental knowledge gaps are concentrated at three interrelated levels: (1) conditional thermodynamics and competitive precipitation in high-ionic-strength multicomponent systems, which constrain the aqueous species and solid phases that are thermodynamically favored and affect the theoretical sulfur demand and selective precipitation sequence; (2) As_2_S_3_ nucleation, growth, and supersaturation evolution under finite mixing and mass-transfer conditions, which determine the characteristics of the particles formed; and (3) the interfacial chemistry and aggregation behavior of As_2_S_3_-rich and mixed-sulfide particles, which affect whether the particles can aggregate effectively and achieve efficient solid–liquid separation. The long-term residue stability discussed in Section 5.4 represents the integrated response of the initial phase composition, particle structure, and interfacial state during subsequent oxidation, dissolution, and aging. Therefore, the four engineering bottlenecks and the three knowledge gaps do not exhibit a simple one-to-one correspondence but instead form a multiscale coupled relationship along the reaction-particle-separation-aging chain. The relationships between these engineering bottlenecks and knowledge gaps are illustrated in Figure 11.

### 6.1. Conditional Thermodynamics and Competitive Precipitation in Multimetal-Sulfide-Water Systems

After sulfide is introduced into HAAW, it simultaneously participates in acid-base equilibria, complexation, redox reactions, gas–liquid partitioning, and solid-phase formation [1,10,11,18,98,99]. The system may contain As(III), As(V), Cu^2+^, Pb^2+^, Zn^2+^, Cd^2+^, Fe(II), and Fe(III), together with anions such as SO_4_^2−^, Cl^−,^ and F^−^ [1,4,12]. Depending on pH, sulfide may occur as H_2_S, HS^−^, and S^2−^. Under strongly acidic HAAW conditions, H_2_S is generally the predominant dissolved S(−II) species, while metal sulfides, elemental sulfur, and other solid phases may also form [10,18,98]. Therefore, sulfide precipitation in HAAW is not governed by a single solubility product but by the combined effects of acid-base equilibria, complexation, redox reactions, gas–liquid partitioning, and solid–liquid equilibria.

The high concentrations of H_2_SO_4_ and dissolved salts in HAAW place the solution in a high-ionic-strength regime, in some cases approaching the mol/L scale [4,12]. Under such conditions, activity coefficients may deviate substantially from unity, and calculations based on ideal-solution or low-ionic-strength assumptions may fail to accurately describe actual species distributions and phase boundaries [122,123]. Models such as the Pitzer approach and specific ion interaction theory (SIT) can account for electrolyte nonideality, but their reliability depends on the availability and quality of ion-interaction parameters and thermodynamic data [122,123]. Consequently, model predictions for complex HAAW depend strongly on the thermodynamic database, activity model, permitted solid phases, and parameter assumptions.

Complexation and competitive reactions further increase the difficulty of prediction. Sulfide species and anions such as H_2_S, HS^−^, SO_4_^2−^, and Cl^−^ can alter aqueous speciation and effective reaction activities of arsenic and coexisting metals [12,18,98]. In addition, differences in sulfide solubility, complexation behavior, and precipitation kinetics among Cu, Pb, Zn, and Cd can alter the instantaneous effective sulfur demand and actual precipitation sequence [6,12,98]. This is an important theoretical reason why sulfide dosage cannot be accurately determined solely from the total arsenic concentration.

The presence of As(V) introduces additional kinetic limitations. Its sulfidation generally involves reduction and, under suitable acidity, redox, and sulfidation conditions, may produce As_2_S_3_-rich arsenic-bearing solids [7,18,99]. Equilibrium thermodynamics can therefore describe only the tendency for phase formation and cannot directly predict the time required for As(V) conversion, the instantaneous sulfur demand, or the actual precipitation sequence. Thus, actual precipitation behavior in HAAW requires simultaneous consideration of activity corrections under high ionic strength, complexation, redox reactions, and competitive precipitation rather than reliance solely on equilibrium solubility products or simple mass balances.

### 6.2. Nucleation, Growth, and Supersaturation Control of As_2_S_3_

Whether As_2_S_3_ ultimately forms relatively large precipitate particles or fine stable colloids depends on the competition between nucleation and particle growth, which is primarily governed by local supersaturation and its spatiotemporal evolution [11,13,101]. Therefore, beyond determining whether As_2_S_3_ is thermodynamically favored, it is necessary to clarify how supersaturation develops, propagates, and decays within the reactor after sulfide addition.

Classical homogeneous nucleation theory indicates that the nucleation rate is highly sensitive to the supersaturation ratio S [116]. For high-ionic-strength HAAW, S should, in principle, be defined on the basis of the activities of the relevant reacting species rather than simple concentrations [122,123]. A generic empirical expression for particle growth as a function of supersaturation can be written as [124]:

G = k_g_ (S − 1)^g^ (19)

where G is the characteristic particle growth rate, k_g_ is the growth kinetic coefficient, and g is an empirical reaction order associated with the specific growth mechanism and system conditions. In the absence of system-specific experimental data, a universal growth exponent should not be assigned to As_2_S_3_ in HAAW. In general, high local supersaturation favors the formation of numerous new nuclei, whereas lower and controlled supersaturation together with a larger available surface area of pre-existing solids favors surface deposition and particle growth.

When soluble sulfide reagents such as Na_2_S or NaHS are introduced into strongly acidic arsenic-containing solutions, sulfide protonation and arsenic sulfidation may occur before complete micromixing is achieved [13,101]. Consequently, the average sulfide concentration in the reactor does not fully represent the local reaction environment near the dosing point. When the characteristic chemical-reaction timescale is substantially shorter than the micromixing timescale, high local supersaturation may induce extensive homogeneous nucleation even when the overall S/As ratio is appropriate. This can explain the observation that arsenic is efficiently converted from the aqueous phase while the resulting particles remain difficult to settle and filter.

Sulfur-source supply mode, dosing strategy, seeding, and reactor mixing can all alter the supersaturation history [10,13,14,101,114]. The essential issue is therefore not simply optimization of the final S/As ratio, but coordination among the sulfur-release rate, mixing rate, chemical-reaction rate, nucleation rate, and particle-growth rate. Figure 12 schematically illustrates the competition between nucleation and growth under different supersaturation conditions.

At present, systematic data on As_2_S_3_ nucleation rates, particle growth rates, and their dependence on pH, temperature, ionic strength, sulfur-source type, and coexisting metal composition under actual HAAW conditions remain limited, restricting quantitative prediction of particle formation.

### 6.3. Interfacial Chemistry and Aggregation of As_2_S_3_-Rich and Mixed-Sulfide Particles

After particle formation, aggregation, settling, and filtration performance are primarily governed by particle interfacial chemistry and interparticle interactions. The aggregation of As_2_S_3_-rich and mixed-metal-sulfide particles is jointly influenced by surface charge, specific ion adsorption, electrical double-layer screening, van der Waals attraction, surface hydration or hydrophobic interactions, hydrolysis products, and surface heterogeneity [13,14,115,117,118,125]. Therefore, solid–liquid separation difficulties cannot be attributed solely to small particle size but should be regarded as the combined result of particle size, surface chemistry, and the actual wastewater matrix.

As_2_S_3_ particles formed by sulfide precipitation are generally negatively charged, and their ζ potential and aggregation behavior are sensitive to solution chemistry [115,125]. Their surface charge and ζ potential are influenced by pH, sulfide speciation, ionic strength, and adsorption of coexisting ions. Although increasing ionic strength generally compresses the electrical double layer, specific adsorption and hydrolysis of multivalent ions may further alter surface potential and, in some colloidal systems, contribute to charge reversal [117,118,126]. Therefore, ionic strength alone cannot accurately predict particle stability.

DLVO theory provides a fundamental framework for understanding the competition between van der Waals attraction and electrical double-layer repulsion, whereas the Schulze-Hardy rule describes the empirical effect of counterion valence on colloidal destabilization trends [117,118,126]. However, the high ionic strength, multivalent ions, surface heterogeneity, and specific adsorption in HAAW may cause actual behavior to deviate from classical DLVO predictions. When an extended DLVO framework is adopted, short-range Lewis acid-base and hydration-related interactions should also be considered [117]. Therefore, these theories are more appropriately used as a mechanistic basis rather than for direct quantitative prediction without system-specific parameterization and experimental validation. Accordingly, Figure 13 schematically illustrates electrical double-layer overlap during particle approach, together with qualitative interaction-energy profiles and electrolyte effects on aggregation within the extended DLVO framework.

In addition, wastewater matrices containing As_2_S_3_-rich particles may also contain metal sulfides such as CuS, CdS, and ZnS; differences among these sulfide phases in surface charge and hydrophilicity/hydrophobicity can further complicate their aggregation behavior [6,98,125]. Although Fe(III)/Al(III) coagulants and polymeric flocculants can improve solid–liquid separation, they also alter the final residue composition. Therefore, the current knowledge gap lies not only in explaining whether particles aggregate, but also in establishing quantitative relationships among interfacial properties → aggregation kinetics → particle-size distribution → solid–liquid separation performance.

### 6.4. Cross-Scale Linkage Between Residue Aging and Long-Term Stability

The long-term stability of the residues represents the ultimate environmental response of the theoretical processes described above. Conditional thermodynamics and competitive precipitation influence the initial phase composition; nucleation and growth affect particle size, crystallinity, and specific surface area; and interfacial chemistry, co-precipitation, and coagulation further modify residue composition and reactive interfaces. These initial solid-phase characteristics collectively influence subsequent oxidation, dissolution, phase transformation, and secondary immobilization.

Because newly formed As_2_S_3_ may be amorphous or poorly crystalline [8,18,127], relying solely on XRD characterization of fresh residues or short-term leaching tests is insufficient for evaluating long-term stability. XRD should be combined with solid-phase characterization techniques such as XPS, Raman spectroscopy, and SEM-EDS, and corresponding analyses before and after aging or leaching should be used to establish relationships among sulfidation conditions → solid-phase composition and structure → aging/oxidation pathways → arsenic release or secondary immobilization [8,120,121,127].

Meanwhile, pH, redox conditions, dissolved oxygen, temperature, and coexisting minerals may all alter residue evolution pathways [8,120,121]. Released arsenic may also be re-immobilized through adsorption onto or reprecipitation with secondary Fe-bearing phases [73,74,75]. Therefore, long-term environmental risk depends on the dynamic competition between “release” and “secondary immobilization” rather than on a single initial phase or one-time leaching concentration [73,74,75,120,121].

In summary, the core issues identified in Section 6 can be expressed as follows: thermodynamics constrains what may form, nucleation and growth determine what type of particles are formed, interfacial chemistry influences how particles aggregate and separate, and these processes collectively shape the long-term environmental behavior of the residues. However, these fundamental insights alone cannot determine the rate-controlling steps or scale-up behavior in actual industrial systems, which must be further examined from the perspectives of reaction engineering and evidence boundaries.

## 7. Critical Discussion and Research Priorities

Section 6 summarizes the fundamental knowledge gaps in HAAW sulfide precipitation. This section focuses on two unresolved engineering-science questions: which processes may limit the overall rate under different sulfur-source conditions, and to what extent existing theoretical and experimental conclusions can be transferred to actual industrial systems. On this basis, priorities for future research are proposed.

### 7.1. Reaction-Engineering Identification of Potential Rate-Limiting Steps

Existing studies demonstrate that sulfide precipitation has a favorable thermodynamic driving force but do not support a single rate-controlling mechanism applicable to all HAAW systems. Classical reaction engineering distinguishes intrinsic chemical reaction, external mass transfer, intraparticle or product-layer diffusion, and reactor mixing as separate processes [128]. In HAAW systems, the physical form of the sulfur source determines the corresponding mass-transfer and reaction pathways.

For soluble sulfide reagents such as Na_2_S/NaHS, rapid protonation and sulfidation can occur before complete micromixing is achieved, and local supersaturation near the dosing point can differ markedly from reactor-average conditions [13,101]. When the chemical reaction is faster than micromixing, local supersaturation and H_2_S release near the dosing point may be more important than the reactor-average conditions [13,101]. Therefore, the sensitivity of early particle formation to dosing location, dosing rate, and agitation conditions can provide an important indication of the influence of micromixing.

For gaseous H_2_S, when chemical-reaction enhancement within the liquid film is not explicitly considered, the volumetric mass-transfer rate from the gas phase to the liquid phase can be approximated as [129,130]:

(20) $$r_{H_{2}S,\mathrm{abs}}=k_{L}a\left(C_{H_{2}S}^{*}-C_{H_{2}S}\right)$$

where k_L_a is the volumetric liquid-side mass-transfer coefficient, $C_{H_{2}S}^{*}$ is the equilibrium dissolved concentration corresponding to the H_2_S partial pressure in the gas phase, and $C_{H_{2}S}$ is the bulk-liquid concentration of dissolved H_2_S. $C_{H_{2}S}^{*}$ should be determined from gas–liquid equilibrium under the relevant temperature, pressure, and electrolyte conditions. When H_2_S consumption by liquid-phase reactions is substantially faster than replenishment by mass transfer, gas–liquid mass transfer may become the principal limitation on the overall sulfur supply [129,130]. Gas flow rate, bubble size, interfacial area, and k_L_a should therefore be considered jointly rather than using gas flow rate alone as an indicator of H_2_S supply efficiency [10,129,130]. When chemical reactions within the liquid film significantly enhance H_2_S absorption, reaction-enhanced mass transfer should also be considered.

The acid dissolution of FeS and ZnS is a typical fluid-particle heterogeneous reaction [16,17,114]. When the particles exhibit a distinguishable unreacted core together with a reaction or passivation layer, the shrinking-core model (SCM) can be used to examine candidate rate-controlling mechanisms [128]. For ideal spherical particles, surface chemical-reaction control and product-layer diffusion control can be expressed as:

1 − (1 − X)^1/3^ = k_r_t (21)

1 − 3(1 − X)^2/3^ + 2(1 − X) = k_d_t (22)

where X is the solid conversion and k_r_ and k_d_ are apparent rate constants. Application of the SCM requires that particle geometry remain approximately preserved, a distinguishable reaction interface be present, and the corresponding mass-transfer or reaction resistance have a physically meaningful interpretation. These relationships should therefore be used as diagnostic tools for candidate mechanisms rather than assigning a controlling step solely on the basis of linear fitting quality. Agitation intensity, particle size, temperature, and conversion-time relationships should be combined with surface and cross-sectional characterization to distinguish the relative contributions of external liquid-film mass transfer, surface reaction, and diffusion/passivation; apparent activation energies may also be used as complementary evidence where appropriate [16,114,128]. Notably, diffusion-controlled kinetic fitting has been reported for FeS-based As(III) removal, whereas S(0)-enriched surface layers have been shown to inhibit FeS dissolution and As(V) removal [16,114]; neither observation establishes a universal controlling mechanism. For highly porous particles or particles undergoing fragmentation or uniform dissolution, grain models, porous-particle models, or other heterogeneous-reaction models that better represent the actual particle structure should be considered [128].

For HAAW containing a high proportion of As(V), the kinetics of the overall As(V) sulfurization/conversion pathway must also be considered. Under some conditions, the formation and transformation of dissolved arsenic–sulfide intermediates may proceed more slowly than sulfide delivery or precipitation, so increasing mixing or mass transfer alone may not substantially shorten the overall residence time [7,18,99,114]. Therefore, identification of rate-controlling steps should incorporate time-resolved As(III)/As(V) measurements rather than relying solely on total As concentrations.

Based on the preceding analysis, Table 8 summarizes the candidate rate-limiting steps, key diagnostic indicators, and corresponding engineering implications for the major sulfide-delivery systems in HAAW treatment.

### 7.2. Evidence Boundaries and Model Transferability

Current evidence reasonably supports the following conclusions: under suitable sulfide activities and redox conditions, the formation of low-solubility arsenic sulfides provides a clear thermodynamic basis for sulfide precipitation in strongly acidic systems [7,18,97,98]; As(III)/As(V) speciation affects theoretical sulfur demand and reaction pathways [7,18,99]; sulfur-source supply mode and local supersaturation are important factors governing particle formation [10,13,101,129,130]; and, in systems with well-defined wastewater compositions and operating conditions, regulation of ORP, pH, and sulfide supply can achieve a certain degree of selective metal separation [6,11,12,107,108,109].

However, several commonly used interpretations are strongly condition-dependent. Tabulated K_sp_ values alone cannot accurately predict the actual precipitation sequence in high-ionic-strength multicomponent HAAW [98,122,123]; ORP set points determined for one system cannot be directly transferred to wastewater with different compositions and operating conditions [11,12,106,107,108,109]; high ionic strength does not necessarily cause rapid destabilization of As_2_S_3_-rich particles [117,118,125,126]; available evidence does not justify assigning a single predetermined rate-controlling step to a sulfur source under all conditions [7,10,16,114,129,130]. In some systems, increased crystallinity may reduce solid-phase reactivity, but crystallinity alone is not a sufficient criterion for long-term environmental stability [8,120,121,127].

Accordingly, transferring laboratory-scale models to industrial systems requires consideration of influent composition, activity corrections, reactor mixing, mass-transfer scale, and solid-phase evolution. Models fitted only to final As removal efficiencies or a single set of experimental conditions cannot be considered universally predictive.

### 7.3. Future Research Priorities

Future research should prioritize issues that directly improve process predictability and scalability. First, thermodynamic databases applicable to high-ionic-strength HAAW should be further developed, and As(V) conversion, sulfur-source release, gas–liquid mass transfer, and competitive precipitation should be incorporated into coupled thermodynamic-kinetic models [7,12,18,99,122,123,129,130]. The principal model variable should evolve from “total sulfur dosage” toward “effective sulfide activity and its spatiotemporal variation.”

Second, the rate-controlling regimes associated with different sulfur sources should be systematically identified. For FeS/ZnS, particle size, agitation, temperature, conversion, and XRD/XPS/SEM characterization should be combined to evaluate external mass-transfer, surface-reaction, and diffusion/passivation control. For H_2_S, emphasis should be placed on obtaining k_L_a, gas-utilization efficiency, and off-gas loss data. For soluble sulfide reagents, the relationship between chemical-reaction and micromixing timescales should be quantitatively determined [13,16,17,101,114,128,129,130].

Third, particle processes should be incorporated into reactor-scale design. Coupling nucleation-growth kinetics and population balance models with CFD/mixing models may extend process prediction from aqueous As concentrations alone to particle-size distributions, particle aggregation, and solid–liquid separation performance [13,101,131].

Fourth, residue stability should be treated as a constraint on process optimization rather than as an independent end-of-pipe evaluation criterion. XRD and complementary solid-phase characterization before and after aging/leaching should be used to establish relationships among process conditions → solid-phase structure → long-term leaching behavior. Depending on the actual phase composition, stabilization routes may include hydrothermal crystallization, oxidative conversion to scorodite or other stable Fe-As phases, and solidification-encapsulation [8,9,19,112,120,121,127].

Finally, before claiming industrial transferability of a model or control strategy, validation should be conducted using actual HAAW and, wherever possible, continuous-flow or pilot-scale systems to evaluate robustness against influent fluctuations, scale-up effects, and long-term operation [101,103]. Selective metal recovery, H_2_S safety, reagent and energy consumption, residue volume, and long-term disposal risks should also be considered comprehensively.

Overall, existing studies have largely established whether efficient arsenic removal can be achieved by sulfide precipitation from HAAW. However, there remains no unified quantitative understanding of which steps limit the actual process rate, how the controlling regime changes with operating conditions, and whether laboratory-scale relationships can be reliably transferred to industrial systems. Section 6 defines the fundamental knowledge gaps underlying these issues, whereas Section 7 provides reaction-engineering criteria and priority research directions. Integrating conditional thermodynamics, mass transfer, particle processes, and long-term stability within a unified framework may facilitate the transition of HAAW sulfide precipitation from empirical operation toward mechanism-driven design and predictable scale-up.

## 8. Conclusions

This review systematically evaluated the major treatment technologies for high-arsenic acidic wastewater (HAAW), with particular emphasis on the reaction fundamentals, engineering bottlenecks, and key theoretical issues associated with sulfide precipitation under strongly acidic conditions. Based on the available evidence, HAAW sulfide precipitation should not be evaluated solely in terms of final arsenic removal efficiency, but rather as a multiscale coupled process spanning the entire reaction-particle-separation-aging chain. Under suitable sulfide activity and redox conditions, sulfide precipitation is thermodynamically feasible and can achieve efficient arsenic removal under strongly acidic conditions without extensive neutralization of the original acidity. Compared with highly neutralization-intensive processes such as lime neutralization, it also offers potential advantages including lower overall sludge generation, preferential enrichment of arsenic in sulfide solids, and selective separation of valuable metals such as Cu. However, its engineering applicability remains constrained by the difficulty in accurately determining sulfide demand, low sulfur utilization efficiency and H_2_S control, poor solid–liquid separation of fine As_2_S_3_-rich particles, and uncertainty in the long-term stability of the resulting residues.

Current evidence supports several relatively well-established mechanistic conclusions. As(III)/As(V) speciation affects sulfide demand and reaction pathways. When rapid sulfidation reactions occur before complete micromixing is achieved, high local supersaturation can promote extensive nucleation and the formation of fine particles. Different sulfur sources follow different reaction and mass-transfer pathways: gaseous H_2_S may be influenced by gas–liquid mass transfer, whereas FeS/ZnS systems involve fluid-particle mass transfer and surface reaction and, where a reaction or passivation layer develops, may also exhibit diffusion resistance. Aggregation and separation after particle formation are further influenced by surface charge, specific ion adsorption, electrical double-layer interactions, and the composition of mixed solid phases. The overall environmental performance is governed not only by aqueous arsenic removal but also by residue phase composition, crystallinity, particle structure, aging behavior, and subsequent disposal conditions. For amorphous or poorly crystalline As_2_S_3_, XRD should therefore be combined with complementary solid-phase characterization and aging/leaching tests to establish relationships between solid-phase structure and long-term stability.

At the same time, several commonly used interpretations remain strongly condition-dependent. Tabulated K_sp_ values alone cannot accurately predict the actual precipitation sequence in high-ionic-strength, multicomponent HAAW. ORP set points obtained for a specific system cannot be directly transferred without considering the reference electrode, solution composition, and operating conditions. Classical DLVO theory cannot, without system-specific parameterization and experimental validation, quantitatively predict particle aggregation in complex, highly concentrated HAAW. Likewise, Na_2_S/NaHS, H_2_S, and FeS/ZnS systems do not share a single rate-controlling step applicable under all conditions. The shrinking-core model has clear mechanistic significance only when the particle structure and reaction interface satisfy its underlying physical assumptions. Accordingly, evaluation of HAAW sulfide precipitation should extend beyond equilibrium results and final removal efficiency to an integrated assessment of thermodynamics, reaction kinetics, mass transfer, particle evolution, and solid-phase stability.

Future research should prioritize the development of thermodynamic databases and coupled thermodynamic-kinetic models applicable to high-ionic-strength HAAW, together with time-resolved measurements of As(III)/As(V), dissolved S(−II) species, and effective sulfide activity. Reaction and mass-transfer limitations under different sulfur-source conditions should be systematically identified, and predictive models coupling micromixing, population balance modeling, and reactor-scale behavior should be further developed. Selective recovery of valuable metals and long-term stabilization of arsenic-bearing residues should also be integrated within a unified process framework, while the transferability of models and control strategies should be validated using actual HAAW in continuous-flow or pilot-scale systems. These advances can facilitate the transition of HAAW sulfide precipitation from empirical overdosing and endpoint control toward mechanism-driven design, predictive control, and whole-process optimization, while simultaneously improving arsenic removal, resource recovery, operational safety, and long-term environmental stability.

## Figures and Tables

**Figure 1:**
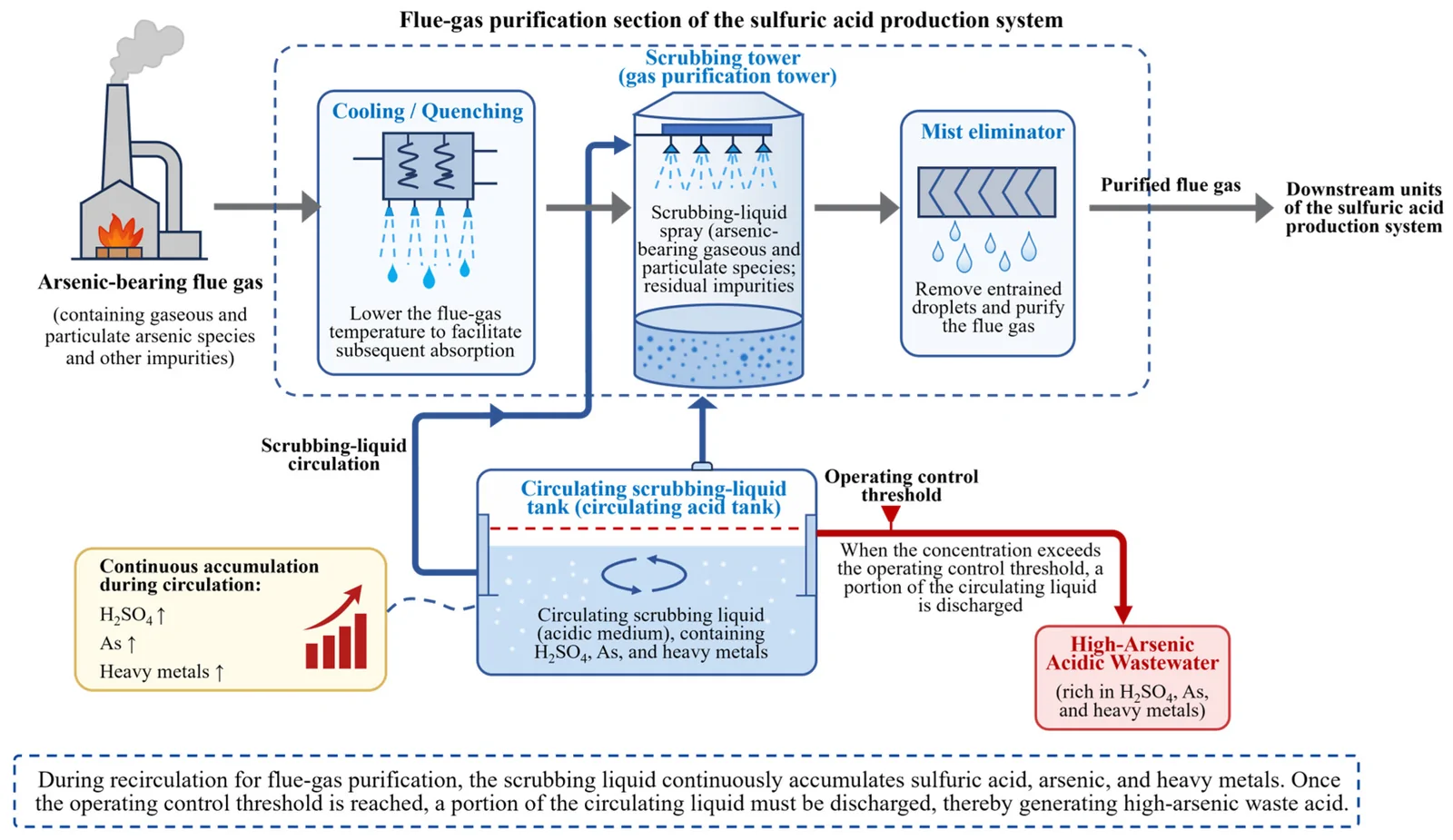
Schematic diagram of HAAW formation in the flue-gas cleaning section of a sulfuric acid plant.

**Figure 2:**
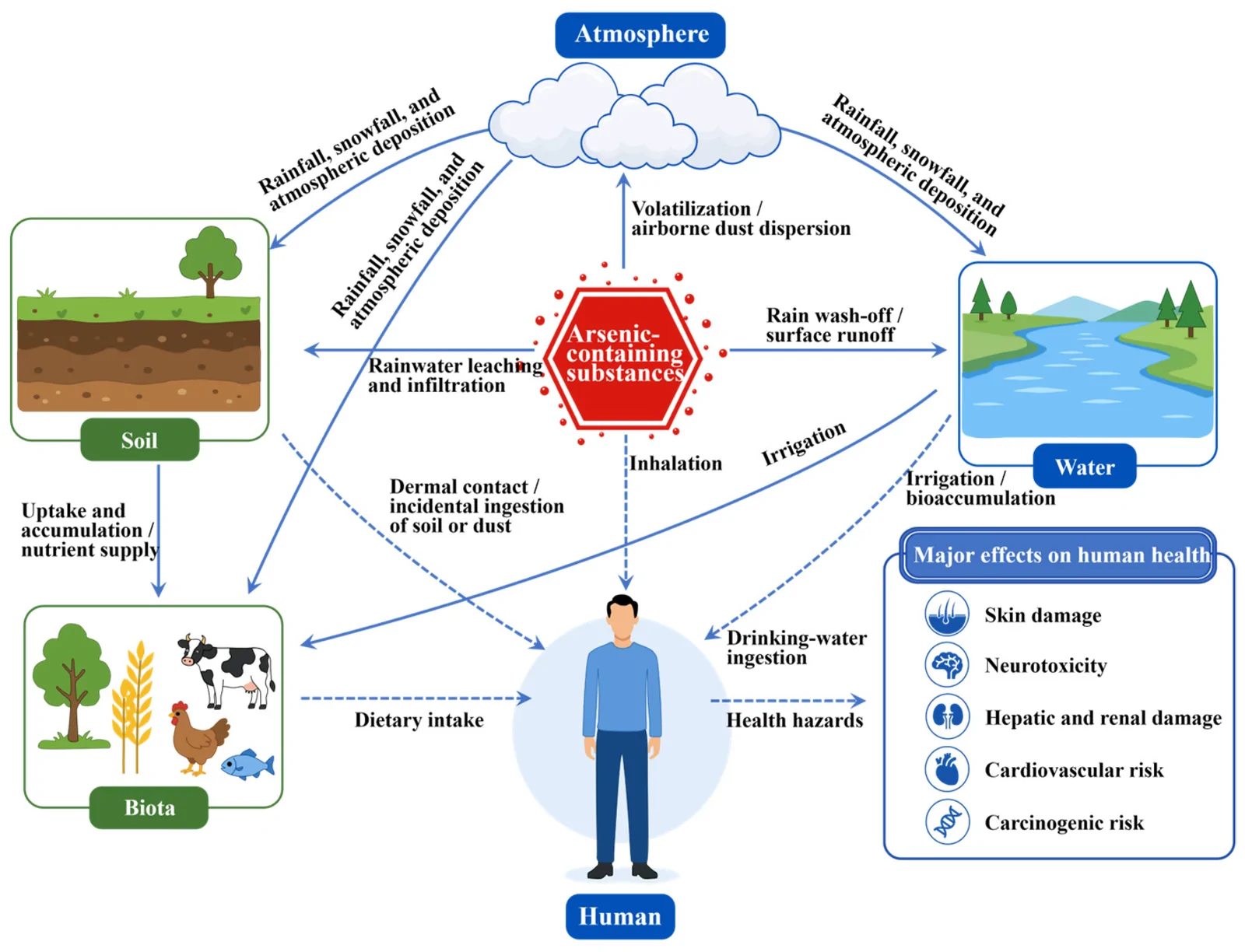
Schematic representation of arsenic migration pathways from industrial sources through environmental media to exposure receptors. Conceptual schematic; pathway importance is site-specific.

**Figure 3:**
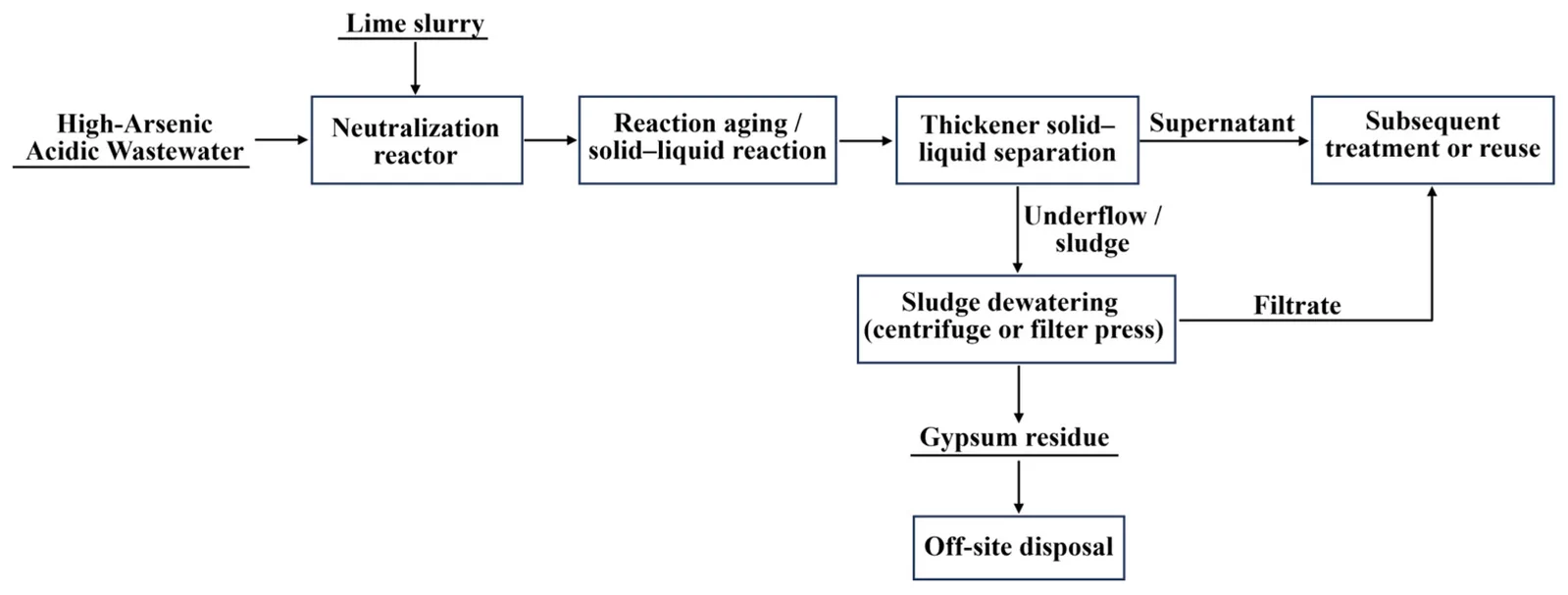
Typical process flow diagram of lime neutralization for HAAW treatment.

**Figure 4:**
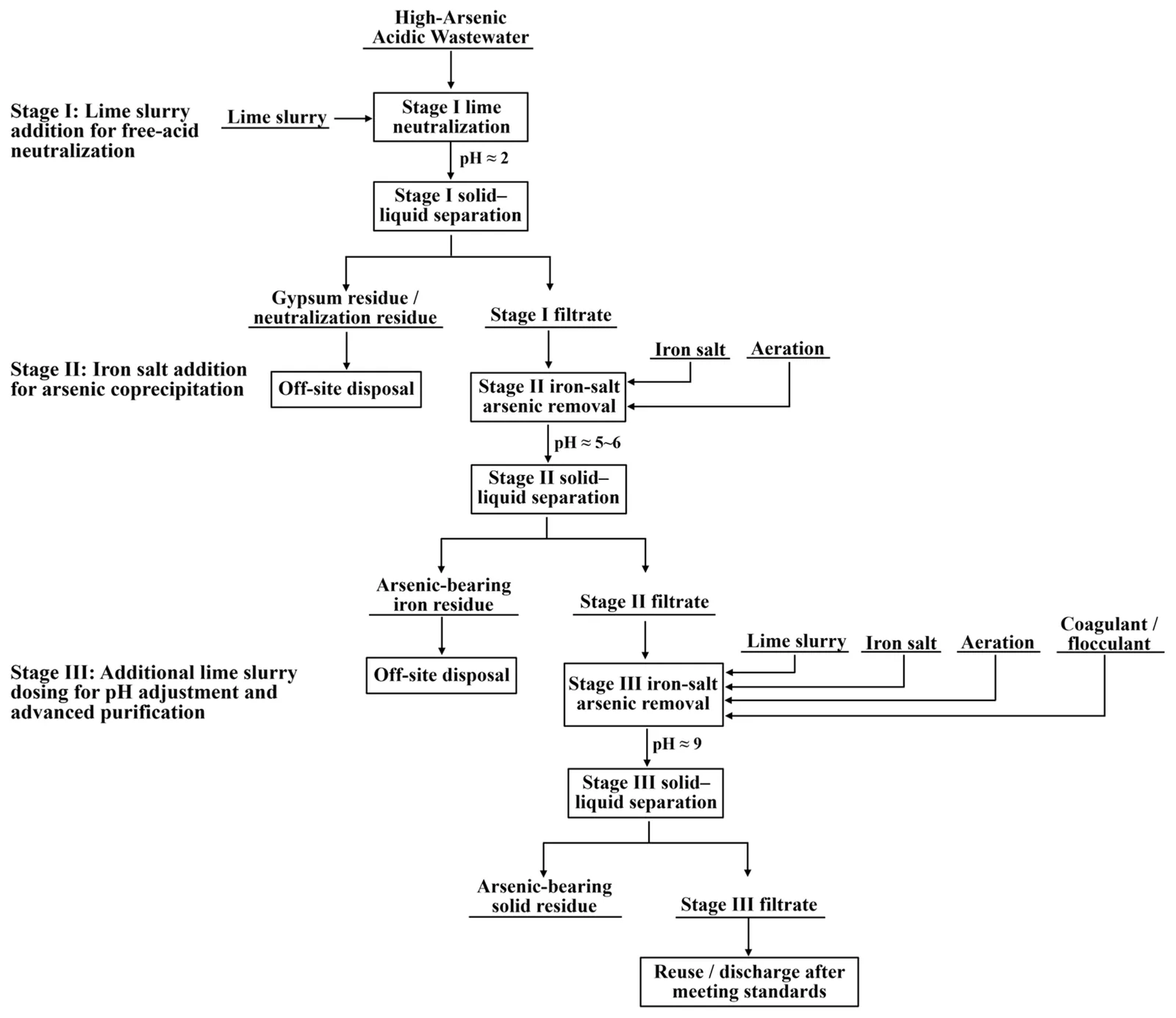
Representative three-stage lime–iron co-precipitation process for HAAW treatment.

**Figure 5:**
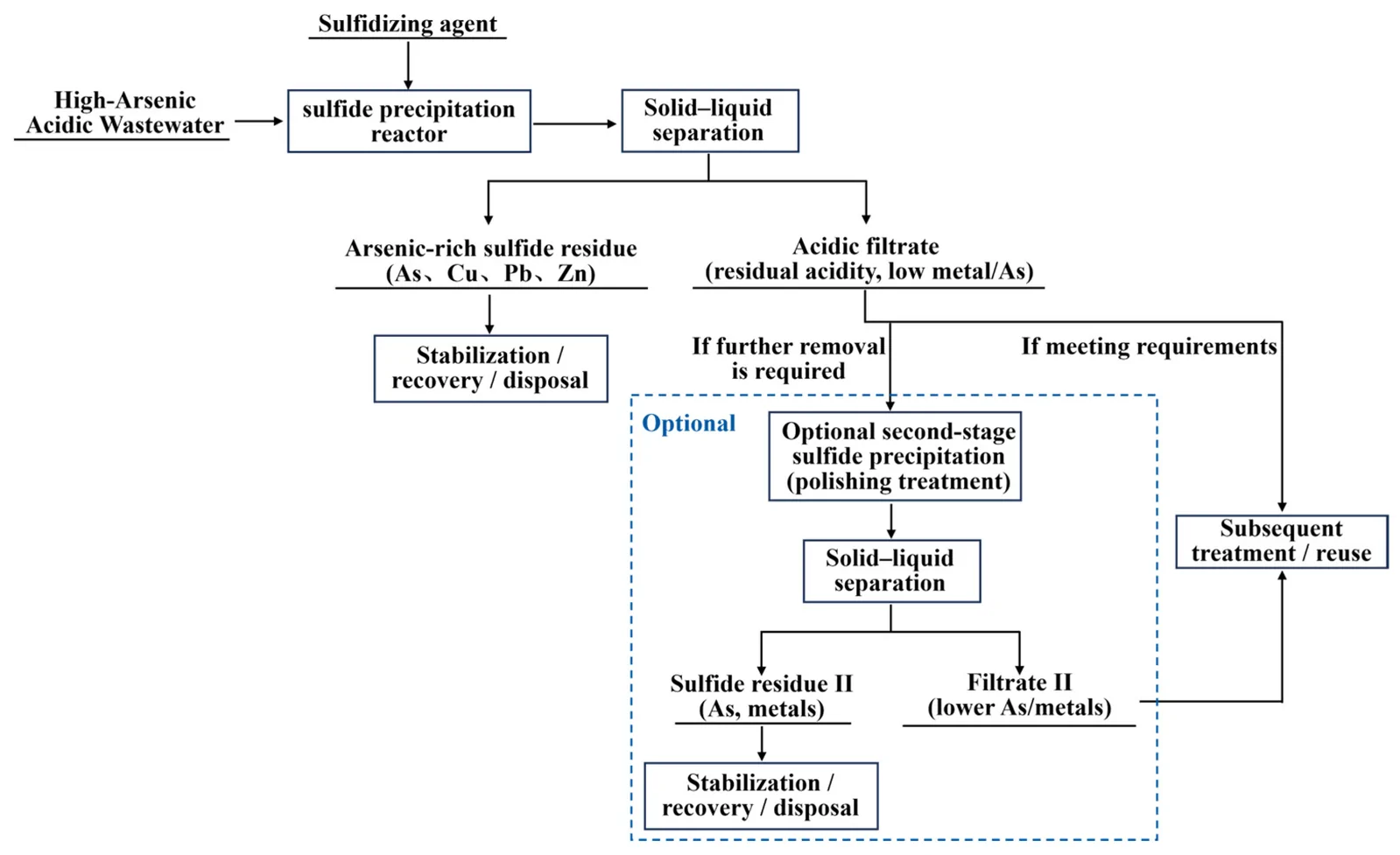
General process flow of sulfide precipitation for HAAW treatment.

**Figure 6:**
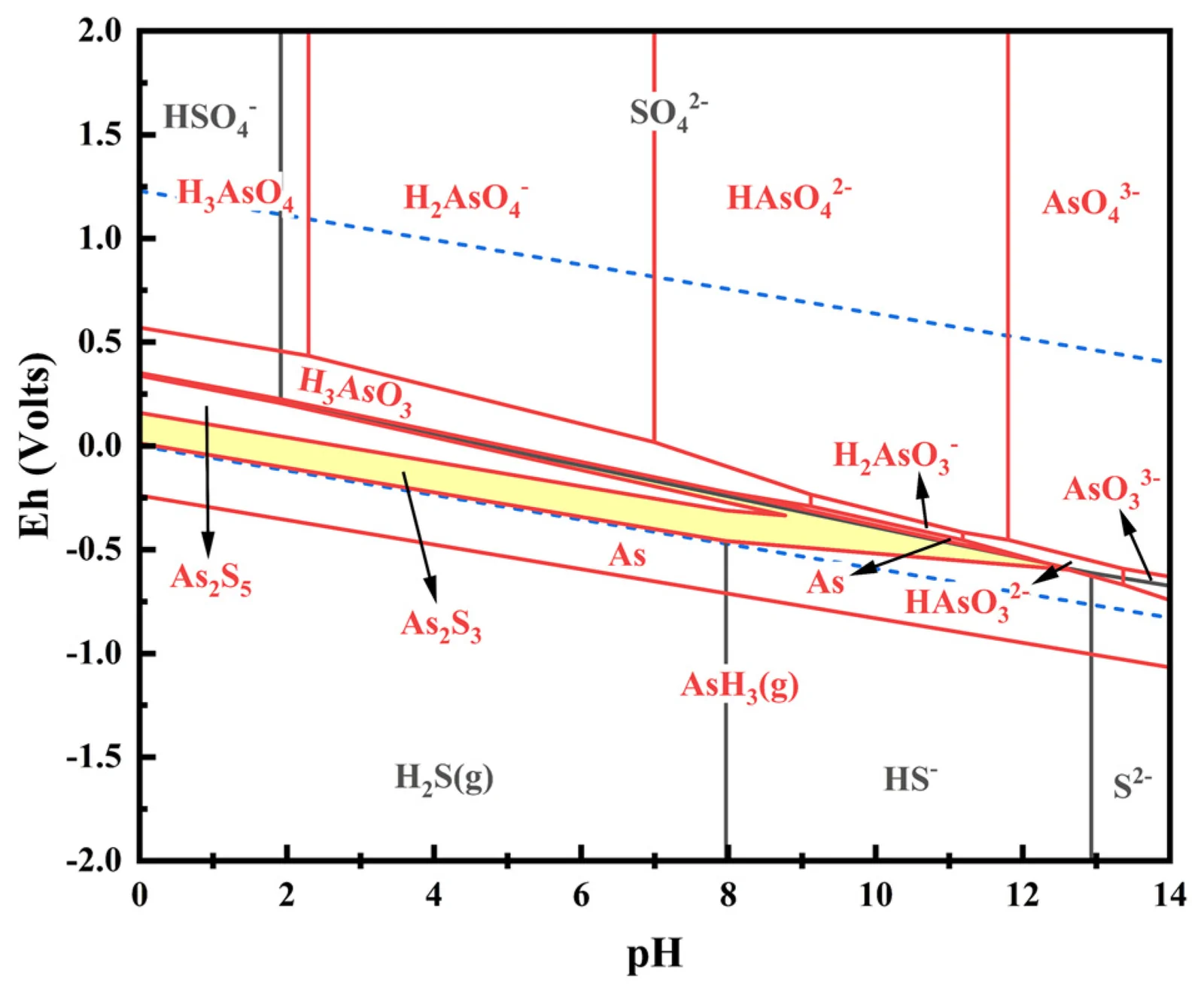
E-pH diagram for the As-S-H_2_O system at 25 °C, calculated with [As] = [S] = 1 mol/L and partial pressures of O_2_ and H_2_ both set to 101,325 Pa (1 atm).

**Figure 7:**
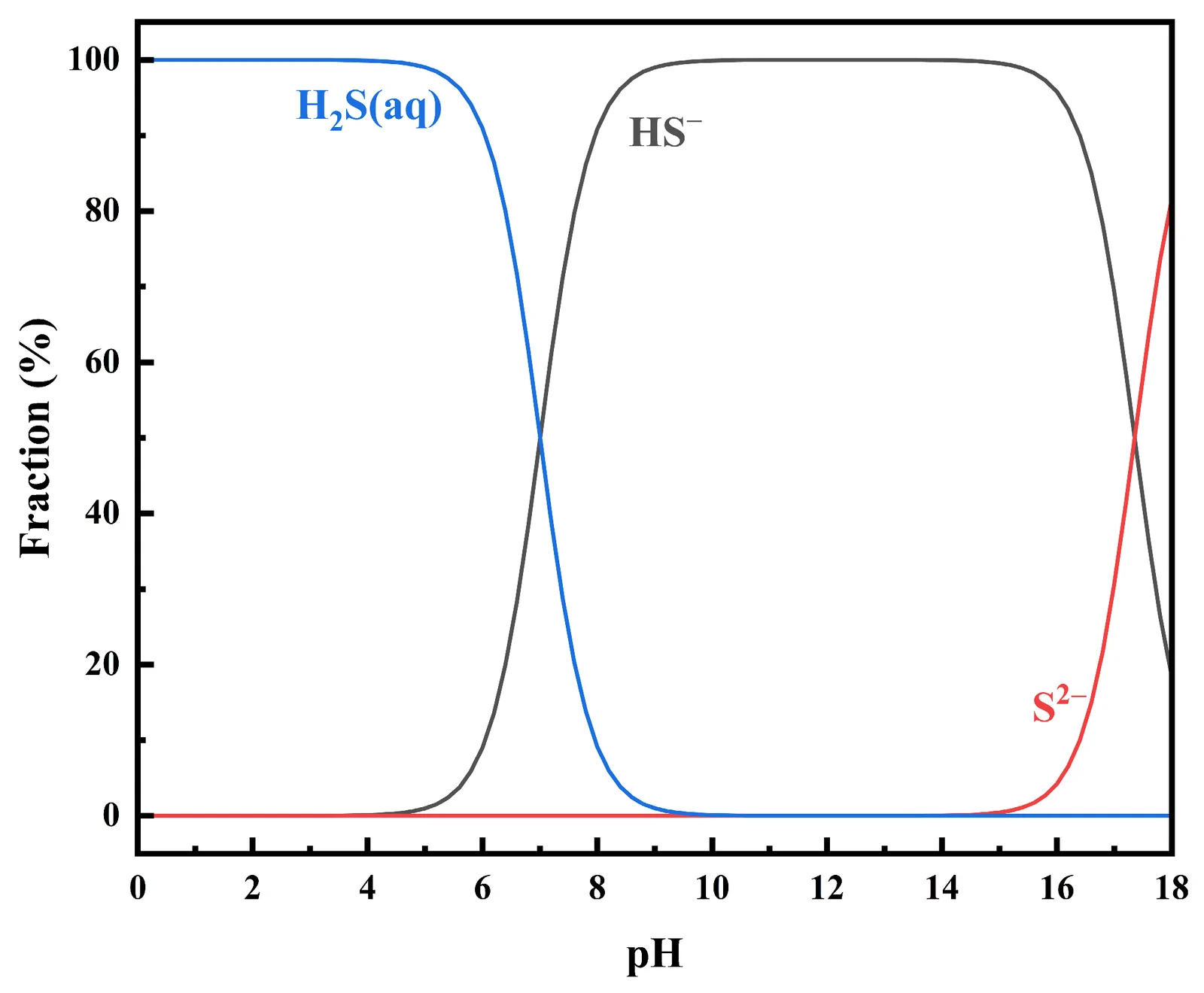
Calculated aqueous sulfide speciation as a function of pH at 25 °C.

**Figure 8:**
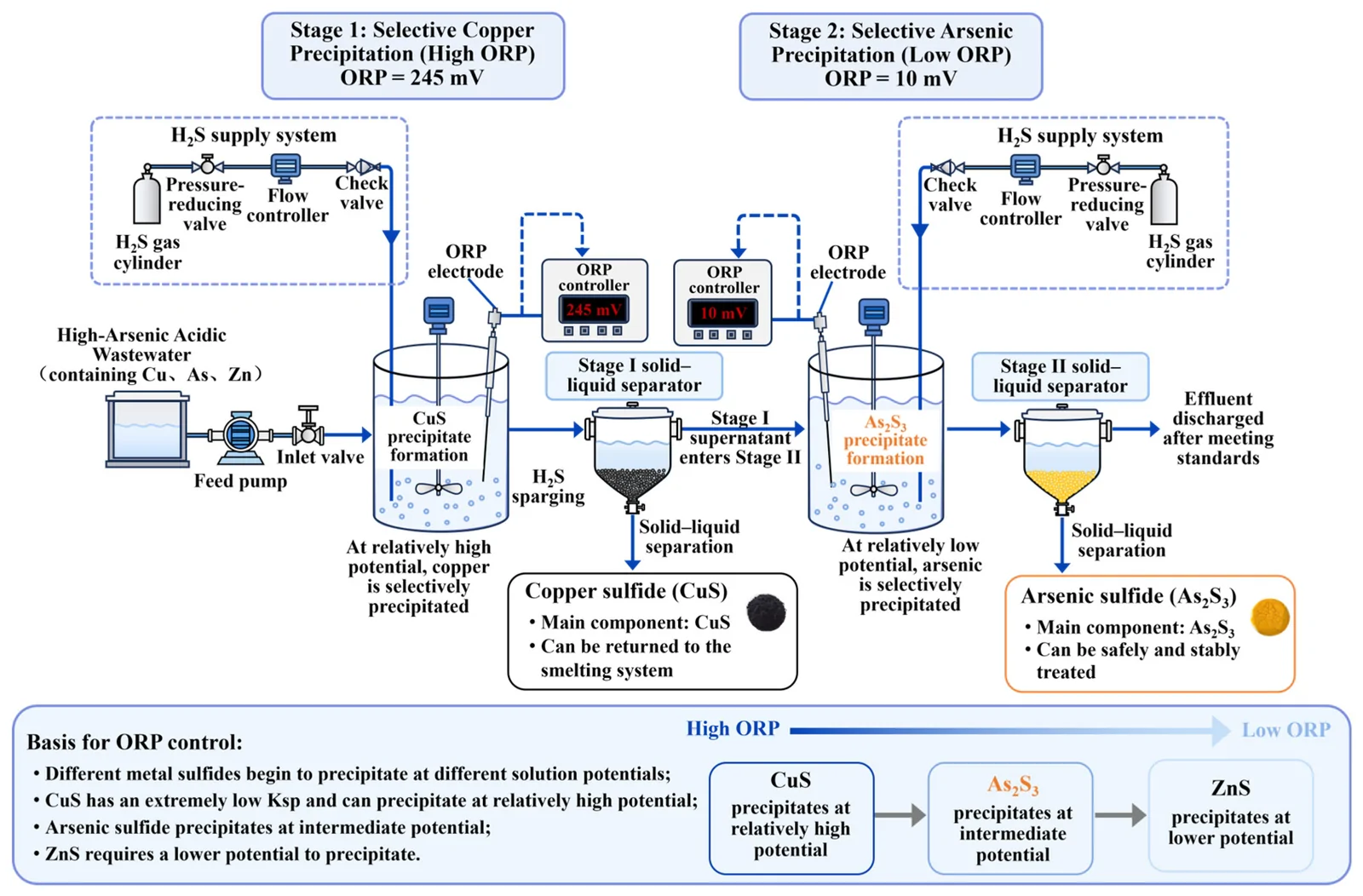
Schematic diagram of two-stage selective sulfide separation by ORP control.

**Figure 9:**
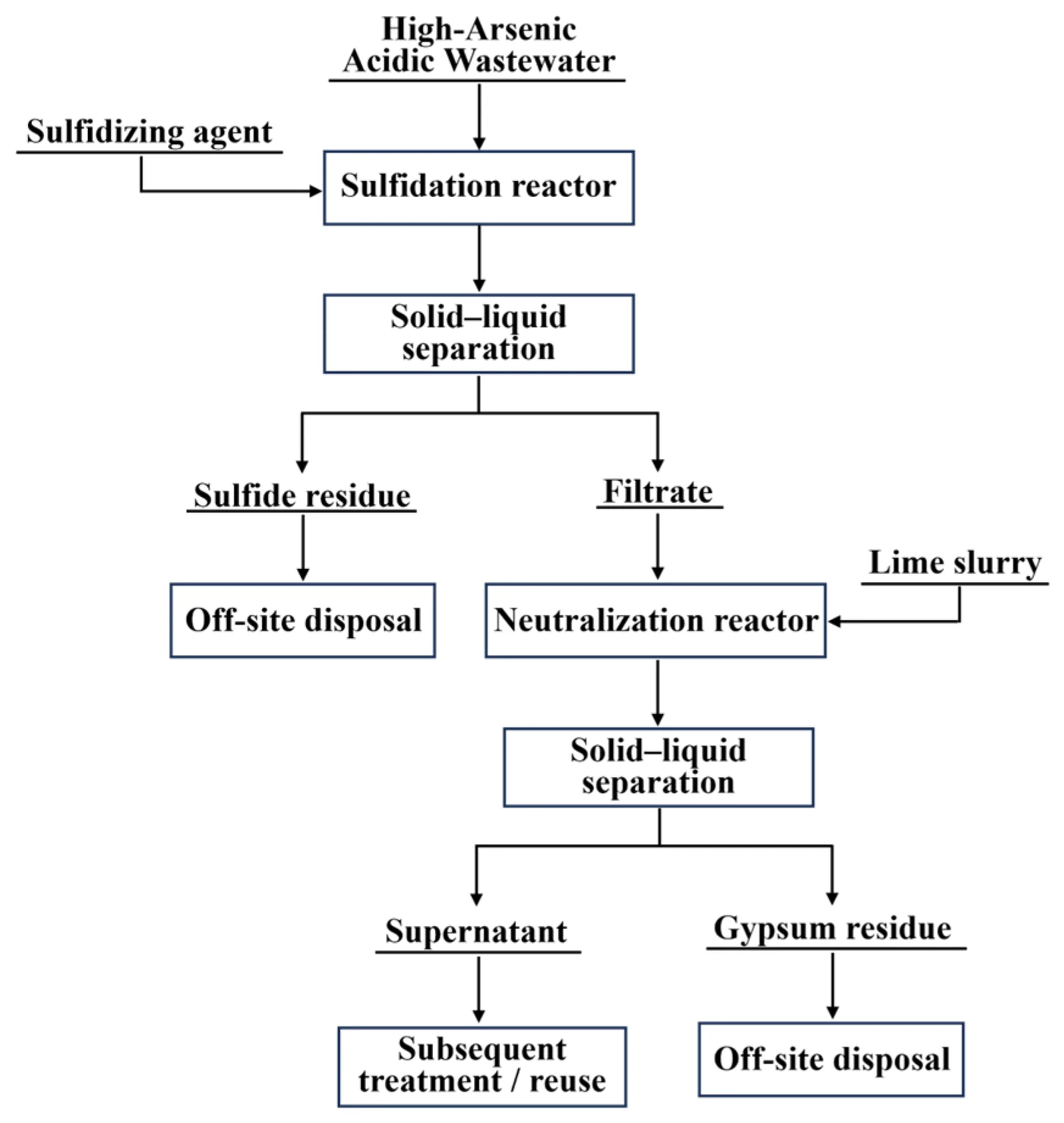
Combined process flow diagram of sulfide precipitation followed by lime neutralization for HAAW treatment.

**Figure 10:**
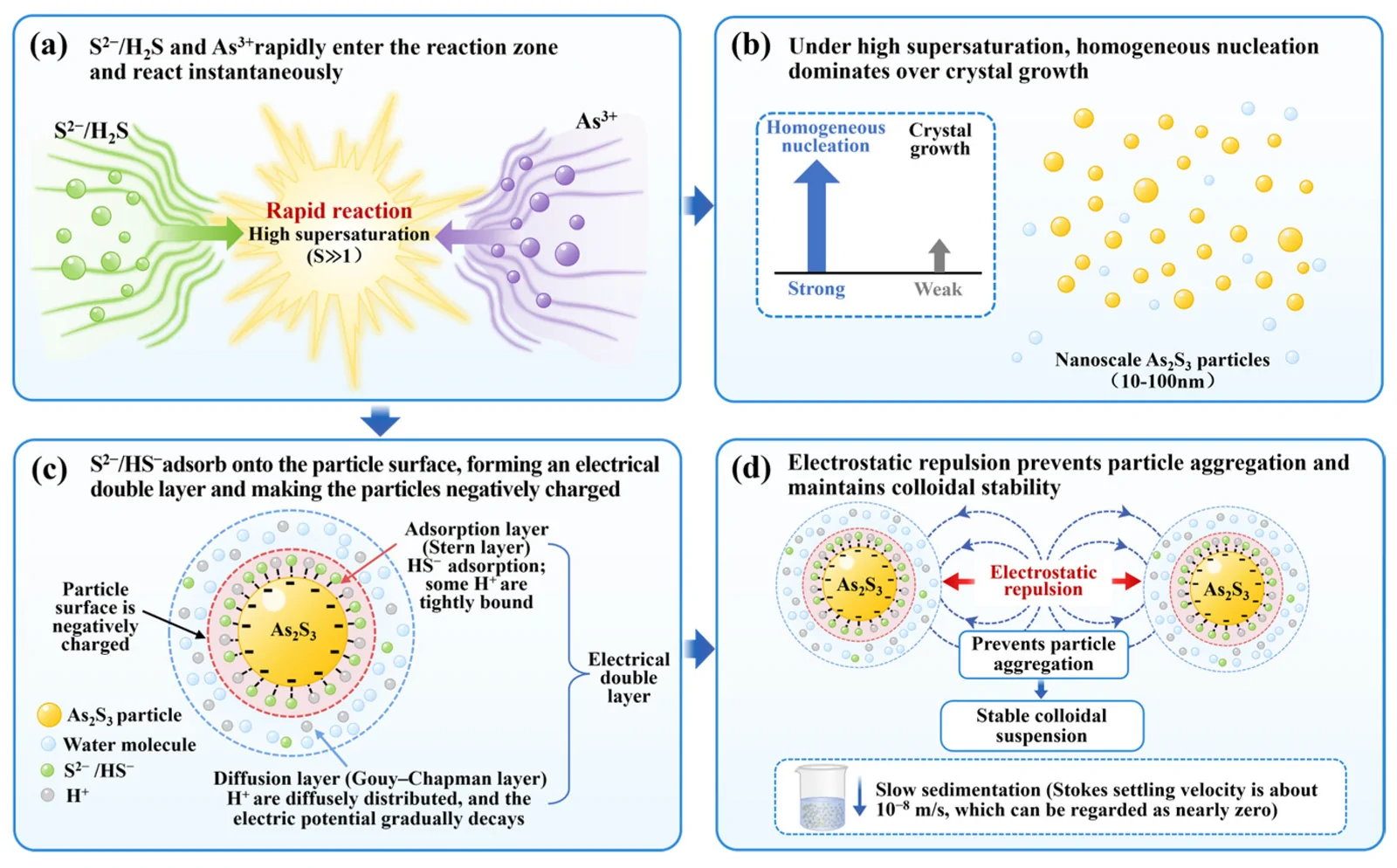
Schematic illustration of colloidal As_2_S_3_ formation and stabilization.

**Figure 11:**
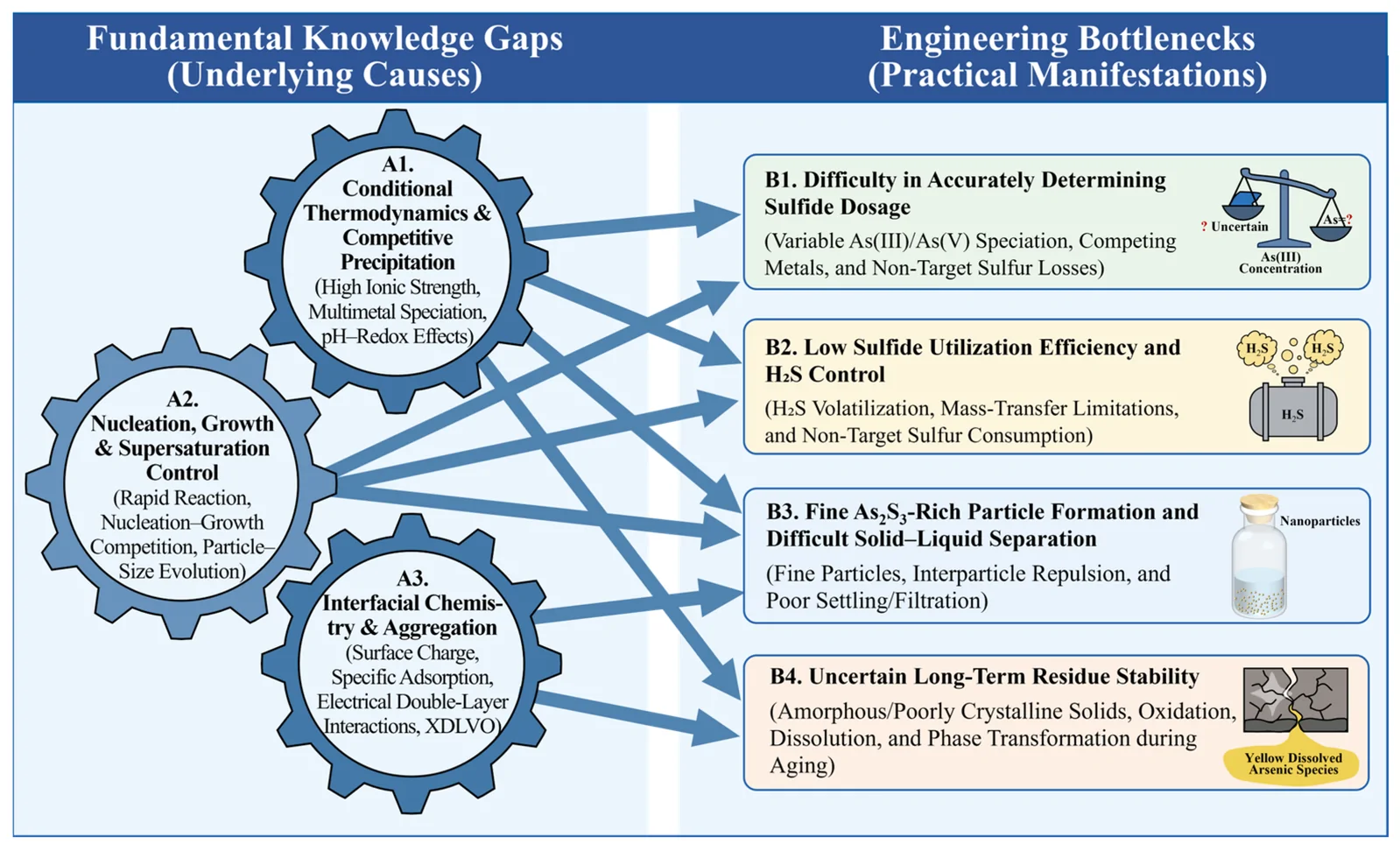
Schematic mapping of the four engineering bottlenecks to three underlying fundamental knowledge gaps.

**Figure 12:**
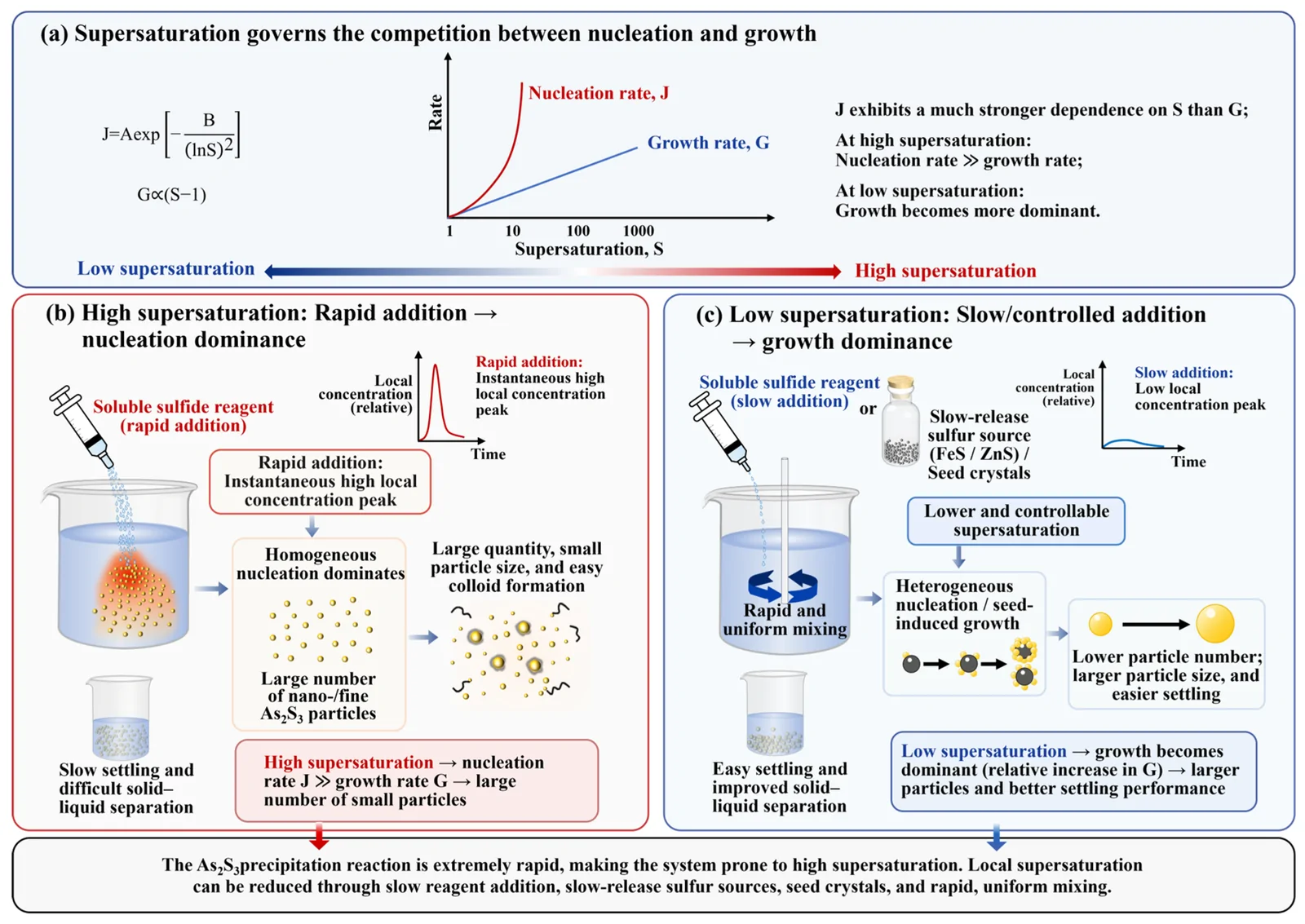
Schematic illustration of nucleation-growth competition as a function of supersaturation.

**Figure 13:**
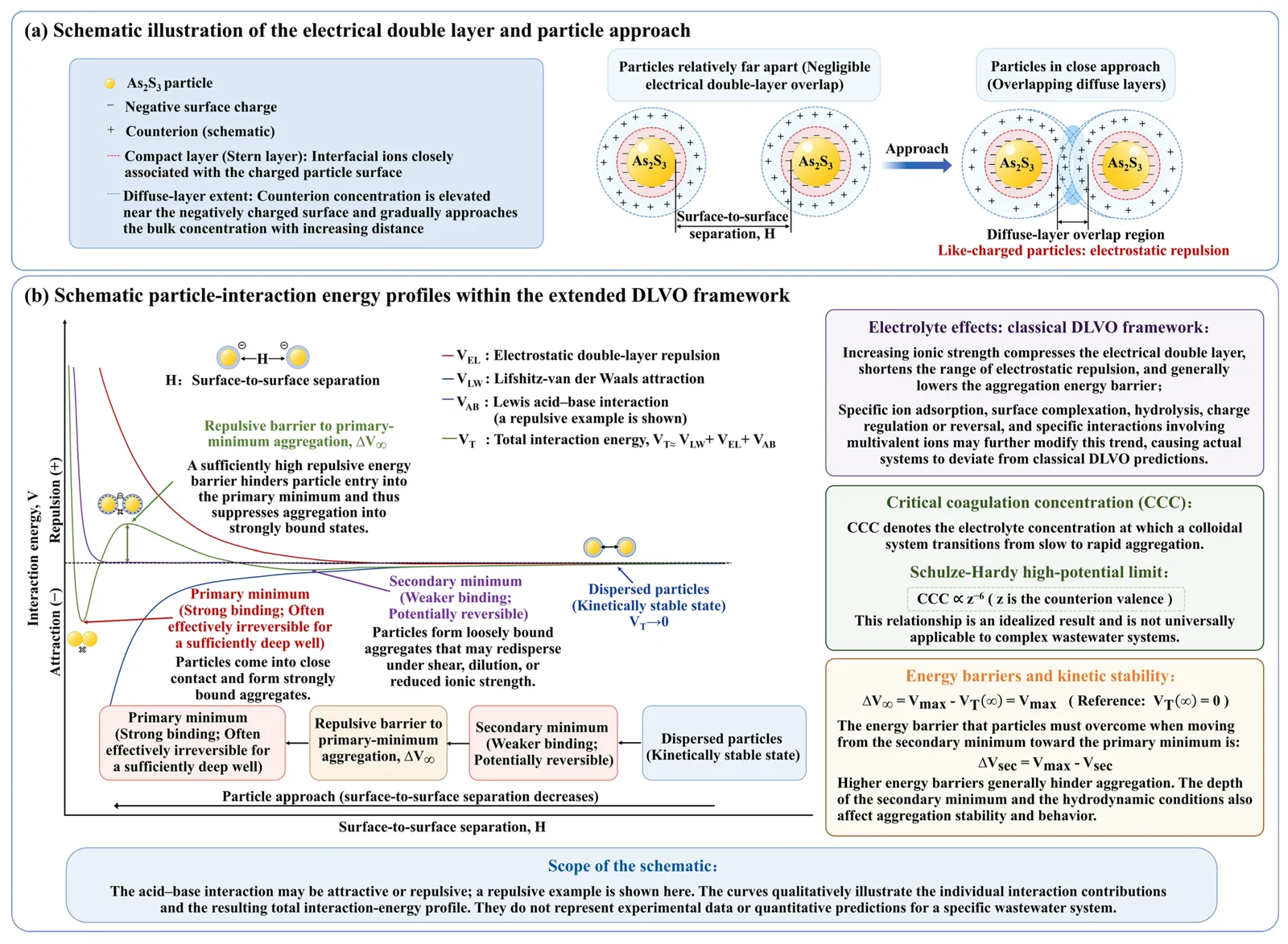
Schematic illustration of (**a**) electrical double-layer overlap during the approach of negatively charged particles and (**b**) qualitative particle-interaction energy profiles and electrolyte effects on aggregation within the extended DLVO framework [117,118].

**Table 1:** Comparison of the scope and emphasis of representative recent reviews with the present review.

Review	Primary Scope	Major Emphasis	Main Scale/Matrix	Distinct Contribution Relative to the Present Review
Estay et al. 2021 [20]	Metal sulfide precipitation	Recent advances and future outlook of sulfide precipitation	Broad metal-bearing waters	Broad sulfide-precipitation perspective; not centered on the full HAAW reaction-to-residue chain.
Zhang et al. 2023 [6]	Selective metal sulfide precipitation	Sulfide agents, operating factors, selectivity, particle aggregation	Broad wastewater systems	Strong treatment of agents/aggregation; the present review adds HAAW-specific reaction engineering, residue aging, and evidence weighting.
Du et al. 2024 [1]	Dirty-acid treatment	Typical industrial technologies, arsenic residues, engineering prospects	Non-ferrous smelting dirty acid	Strong process/engineering overview; the present review focuses more deeply on mechanistic coupling behind sulfide bottlenecks.
Hong et al. 2025 [5]	Cu-As polymetallic acidic wastewater	Sulfide sources, operating factors, selective separation	Acidic Cu-As wastewater	Focused sulfide review; the present review additionally integrates nucleation, colloid interfaces, XRD/phase evolution, and long-term residue stability.
Liu et al. 2026 [12]	Cu/As separation in copper-smelting waste acid	Separation strategies, competitive sulfidation mechanisms, monitoring perspectives	Copper-smelting waste acid	Detailed Cu/As separation focus; the present review addresses broader HAAW treatment and the reaction-particle-separation-aging chain.
Present review	HAAW treatment and stabilization	Four engineering bottlenecks linked to three knowledge gaps; reaction engineering; particle formation; XRD and residue stability	Strongly acidic multicomponent HAAW, emphasizing real/pilot/industrial evidence	Integrates chemistry, transport, particle science, solid characterization, separation, and residue aging into one mechanistic framework.

The comparison is qualitative and intended to clarify scope rather than rank review quality.

**Table 2:** Reported leaching characteristics and solubility products of representative calcium arsenate phases [60,64,66].

Formula	pH Value of Leachate	Arsenic Concentration in Leachate (mg/L)	K_sp_
CaHAsO_4_	Neutral	1703–2191	—
CaHAsO_4_·H_2_O	Neutral	2025–2408	—
Ca_3_(AsO_4_)_2_	Neutral	95.4	10^−20.96^
Ca_3_(AsO_4_)_2_·H_2_O	7.53	606.9–790.4	—
Ca_3_(AsO_4_)_2_·2.25H_2_O	7.41	2144	10^−21.40^
Ca_3_(AsO_4_)_2_·3H_2_O	5.15	3464	10^−21.14^
Ca_5_(AsO_4_)_3_OH	9.78	27.72	10^−40.12^
Ca_4_(OH)_2_(AsO_4_)_2_·4H_2_O	13.4	0.44	10^−27.49^

**Table 3:** Typical process conditions and performance of lime-iron co-precipitation processes.

Process Type	Conditions	Initial As (mg/L)	Effluent As (mg/L)	Removal Rate	Reference
Two-stage lime–Fe(III) co-precipitation	Continuous lime–Fe(III) co-precipitation; Fe/As = 4; pH 4 → pH 8	1418	0.008	>99.99%	[80]
Three-stage CaCO_3_-Ca(OH)_2_-Fe(III) process	CaCO_3_ pretreatment → Ca(OH)_2_ neutralization → Fe(III) polishing	—	<0.2	—	[70]
Three-stage lime–ferrous iron process	pH 2 → pH 5–6, Fe/As = 4:1, FeSO_4_ + aeration → pH 9, Fe/As = 4:1, FeSO_4_ + aeration	14,740	0.25	>99.99%	[76,77]
Optimized lime–iron salt process	pH 1.2, 2 h → H_2_O_2_ oxidation, pH 8, Fe/As = 4.5, 3 h	High	0.12	~100%	[79]

**Table 4:** Typical process conditions and performance of scorodite precipitation processes.

Process Type	Fe/As Ratio	Temperature (°C)	pH	Reaction Time	As Precipitation (%)	As Leaching (mg/L)	Reference
Hydrothermal (optimized)	1.0	150	1.5	5 h	—	0.08	[86]
Hydrothermal	0.5–3.0	105–175	1.0–2.0	—	>80	<5	[86]
Atmospheric	—	95	0.3–1.0	1–7 h	—	<0.1	[88,89]
Multi-stage	—	70	1.1 → 3.8	5 × 24 min	97.1	—	[90]

The residue endpoint differs among studies (leaching result, phase identity, or metal loss); entries should not be interpreted as a single standardized ranking.

**Table 5:** Comprehensive comparison of major HAAW treatment and stabilization technologies.

Technology	Representative As Removal Performance	Acidity Preservation	Sludge/Residue Characteristics	Residue Stability and Final Environmental Risk	Selective Recovery Potential	Maturity and Applicable Scenarios
Lime neutralization [1,59,67,68,69]	Moderate to relatively high under multistage treatment	Poor; acidity is neutralized	Large quantities of gypsum-rich and Ca–As-bearing sludge	Relatively low and susceptible to aging and phase transformation, with potential remobilization risk	Low	Mature process; suitable for acidity neutralization and subsequent treatment
Lime-ferric co-precipitation [70,76,79,81,82]	Generally higher than lime treatment alone	Poor	Large quantities of gypsum- and iron-containing sludge	Moderate; affected by Fe-As phase composition, crystallinity, and aging	Low-moderate	Mature process; suitable for advanced arsenic removal and integrated treatment trains
Scorodite precipitation [78,83,84,85,86,87,88,89,90]	High arsenic removal under controlled crystallization conditions	Relatively high; operates under acidic conditions	Relatively small amounts of arsenic-rich crystalline solids	High when well crystallized, with relatively low leaching risk, but dependent on synthesis and storage conditions	Low-moderate	Higher process complexity; suitable where long-term stabilization is prioritized
Sulfide precipitation [1,6,9,11,20,23]	Reported to reach >99% in optimized high-arsenic acidic systems	High; can operate without complete neutralization	Relatively low sludge volume and high arsenic grade, but fine particles or colloids may form	Moderate and condition-dependent; As_2_S_3_ may undergo oxidation and phase transformation	High; potential for staged Cu/As separation	Supported by industrial and pilot-scale applications; suitable for high-concentration, strongly acidic HAAW
Adsorption/membrane separation/ion exchange [15,92,93,94]	High removal can be achieved for suitable low-load influents	Process-dependent	Spent adsorbents, regenerant solutions, or concentrates	Dependent on subsequent management of spent media and concentrates	Limited	Mainly used for advanced treatment after bulk contaminant removal
Biochemical methods [95,96]	Effective arsenic removal can be achieved under suitable low-concentration conditions	Generally requires conditions compatible with biological activity	Biomass or biosorbent residues may require subsequent management	Dependent on arsenic transformation pathways and subsequent residue management	Limited	Primarily suitable for lower-strength arsenic-bearing streams; limited applicability to raw HAAW

Because feed composition, initial arsenic concentration, operating conditions, and evaluation methods differ among studies, the reported arsenic-removal efficiencies are not directly comparable. Accordingly, these data are intended primarily to illustrate the representative treatment performance and engineering characteristics of the different technologies.

**Table 6:** Representative reported solubility-product constants of selected metal sulfides and As_2_S_3_ [5,20].

Sulfide	CuS	PbS	As_2_S_3_	CdS	ZnS	FeS
Representative K_sp_	10^−36^	10^−28^	10^−29^	10^−27^	10^−24^	10^−19^

**Table 7:** Representative performance of different sulfide reagents for arsenic removal from HAAW.

Sulfide Reagent	Representative Conditions	Initial As (mg/L)	Removal Rate (%)	Effluent As (mg/L)	Main Advantages	Main Limitations	Reference
Na_2_S·9H_2_O	20 °C; 1.2× theoretical dosage; 15 min dosing; 15 min reaction; PAM flocculation	16,060	>99.99	0.23	Rapid reaction; easy operation	Na^+^ introduction; risks of H_2_S release and local supersaturation	[102]
NaHS (two-stage, ORP-controlled)	10 wt.% NaHS; second-stage ORP ≈29–30 mV; 55 mL/2 L first-stage filtrate; mixing >2 min	2731	99.12	24	Rapid arsenic precipitation; selective Cu/As separation; production-scale validation	Requires staged ORP control; Na^+^ introduction; excessive dosing increases H_2_S generation	[105]
H_2_S (two-stage)	1920 m^3^/d; continuous gas supply	4500	≈99.8	<10	No Na^+^ introduction; adjustable sulfur supply	Toxic gas handling; affected by gas–liquid mass transfer	[103]
H_2_S (strongly acidic system)	500 ppm H_2_S; 400 mL/min; 30 °C; 3 h	500	99.87	0.28	High arsenic removal efficiency; no Na^+^ introduction	High corrosion and safety requirements	[97]
Monoclinic FeS	Acidity = 120 g/L; S/As = 3.75; 40 °C; 150 min	1889	99.92	1.54	Controlled sulfur release; favorable for heterogeneous reactions	Longer reaction time; Fe introduction; possible surface passivation	[16]
ZnS (ultrasound-assisted)	Acidity = 120 g/L; S/As = 3:1; 60 °C; 100 W; 120 min	1017	99.96	0.42	Controlled sulfur release; ultrasound-enhanced mass transfer	Additional energy input; Zn introduction	[17]

The influent composition, As concentration and oxidation state, temperature, basis for S/As calculation, residence time, and solid–liquid separation method differ among studies. Therefore, the above data are intended only to illustrate the representative performance and engineering characteristics of different sulfur sources and should not be used for direct performance ranking.

**Table 8:** Reaction-engineering framework for diagnosing potential rate-limiting steps in HAAW sulfide precipitation.

Sulfur Source/System	Potential Rate-Limiting Steps	Key Diagnostic Indicators	Engineering Implications
Na_2_S/NaHS	Micromixing, rapid reactions, and H_2_S release	Dosing location/rate, agitation sensitivity, early particle-size evolution	Optimize dosing and micromixing
H_2_S(g)	Gas–liquid mass transfer and liquid-phase reactions	k_L_a, bubble size, gas holdup, off-gas/dissolved H_2_S	Increase interfacial area and gas utilization efficiency
FeS/ZnS	External liquid-film mass transfer, surface reaction, diffusion/passivation	Agitation, particle size, temperature, X-t curves, and solid-phase characterization	Distinguish controlling mechanisms and optimize sulfur release
HAAW containing a high proportion of As(V)	As(V) sulfurization/conversion, including intermediate-species transformation	Time-resolved As(III)/As(V); intermediate-species analysis where feasible	Total As alone cannot determine the required residence time

The steps listed in Table 8 should be regarded as candidate rate-limiting mechanisms requiring experimental validation; multiple resistances may coexist or shift in relative importance as the reaction proceeds.

## Data Availability

No new data were created or analyzed in this study. Data sharing is not applicable to this article.
